# Carbon-Induced Changes in the Morphology and Wetting Behavior of Ionic Liquids on the Mesoscale

**DOI:** 10.1021/acs.langmuir.4c00102

**Published:** 2024-02-12

**Authors:** Rita M. Carvalho, Luís M. N. B. F. Santos, Margarida Bastos, José C. S. Costa

**Affiliations:** CIQUP, Institute of Molecular Sciences (IMS), Department of Chemistry and Biochemistry, Faculty of Science, University of Porto, Rua do Campo Alegre s/n, P4169-007 Porto, Portugal

## Abstract

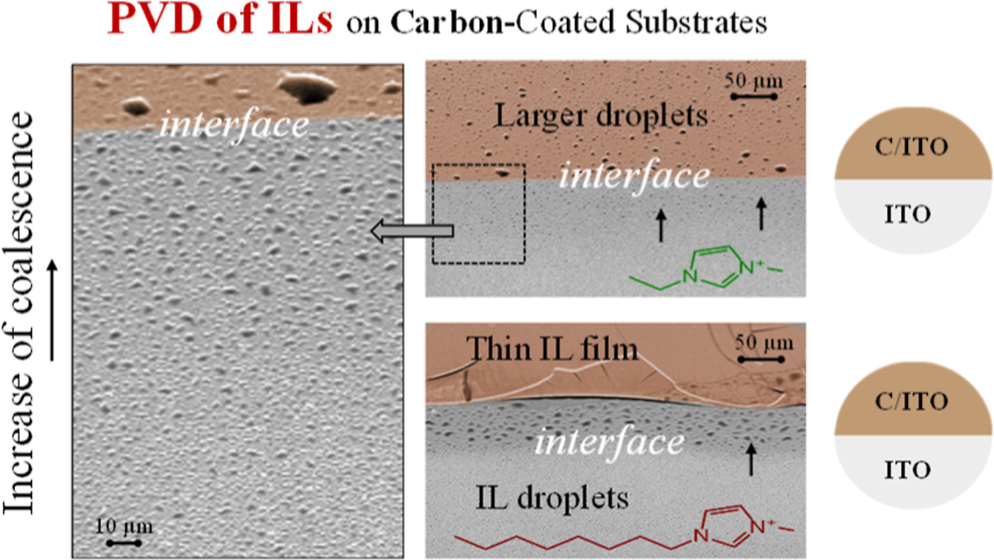

Thin films of ionic liquids (ILs) have gained significant attention due to their unique properties and broad applications. Extensive research has focused on studying the influence of ILs’ chemical composition and substrate characteristics on the structure and morphology of IL films at the nano- and mesoscopic scales. This study explores the impact of carbon-coated surfaces on the morphology and wetting behavior of a series of alkylimidazolium-based ILs. Specifically, this work investigates the effect of carbon coating on the morphology and wetting behavior of short-chain ([C_2_C_1_im][NTf_2_] and [C_2_C_1_im][OTf]) and long-chain ([C_8_C_1_im][NTf_2_] and [C_8_C_1_im][OTf]) ILs deposited on indium tin oxide (ITO), silver (Ag), and gold (Au) substrates. A reproducible vapor deposition methodology was utilized for the deposition process. High-resolution scanning electron microscopy, atomic force microscopy, and X-ray photoelectron spectroscopy were used to analyze the morphological and structural characteristics of the substrates and obtained IL films. The experimental data revealed that the IL films deposited on carbon-coated Au substrates showed minor changes in their morphology compared to that of the films deposited on clean Au surfaces. However, the presence of carbon coatings on the ITO and Ag surfaces led to significant morphological alterations in the IL films. Specifically, for short-chain ILs, the carbon film surface induced 2D growth of the IL film, followed by subsequent island growth. In contrast, for long-chain ILs deposited on carbon surfaces, layer-by-layer growth occurred without island formation, resulting in highly uniform and coalesced IL films. The extent of morphological changes observed in the IL films was found to be influenced by two crucial factors: the thickness of the carbon film on the substrate surface and the amount of IL deposition.

## 1 Introduction

In recent years, there has been extensive research on the properties and applications of ionic liquid (IL) films at the nano- and mesoscale levels. This research interest stems from the remarkable properties exhibited by ILs when used as interfacial materials.^1−7^ One notable characteristic of most ILs is their low volatility, making them a unique class of compounds exceptionally suitable for a wide range of vacuum applications.^8−17^ ILs exhibit exceptional thermal and chemical stability, being in the liquid state across a wide temperature range, retaining their structural integrity.^18−22^ Moreover, ILs demonstrate remarkable solvating properties and can serve as solvents and electrolytes, even under vacuum conditions.^23−28^ The chemical structure of ILs can be tailored to meet specific requirements related to various properties, such as density, viscosity, surface tension, and adhesion strength.^29−33^ Moreover, by careful selection of cations and anions in the studied ILs, customizable surface and interfacial properties can be obtained, allowing precise control over wettability, surface energy, and other related characteristics.^34−40^ These exceptional properties make ILs highly desirable for coating applications.^40−44^

Extensive research has been conducted to understand the interactions between IL films and coatings obtained by vapor deposition on different solid substrates. Particularly, studies have been carried out at the nanoscale level to gain insight into the adsorption and structural configuration of the first monolayers (MLs) of an IL on many surfaces, mainly on metals.^2,10,13,45−55^ Furthermore, studies focusing on the mesoscopic scale have been undertaken to examine the impact of the IL’s chemical structure and the nature of the support on the film’s morphology. The influence of cations, anions, and the length of the alkyl chain on film wetting and morphology has been investigated. Additionally, the effects of deposition rate, substrate temperature, and roughness on nucleation, droplet formation, and spreading during the film deposition process have all been addressed.^1,8,9,12,56−67^ The nucleation and growth mechanisms of IL films fabricated through thermal evaporation are primarily governed by three key factors: the surface diffusion of ion pairs, the availability of a minimum free area to promote nucleation, and the subsequent coalescence processes.^58^ These processes are significantly influenced by the affinity of the ion pairs to the substrate surface as the surface diffusion of ion pairs, driven by their search for favorable binding sites, plays a crucial role in the initial stages of film formation. The presence of a minimum free area is essential for nucleation, allowing the ions to aggregate and form the first stable clusters or *nuclei*. As the nucleated clusters increase in size, they tend to merge with the neighboring clusters, resulting in the growth of the film. The affinity between the ion pairs and the substrate surface strongly influences the coalescence behavior as stronger adhesion promotes the formation of a continuous film, with layer-by-layer [two-dimensional (2D)] growth, while weaker adhesion leads to the formation of discrete islands (three-dimensional (3D) growth).^68−71^ Numerous studies have consistently reported the formation of a 2D wetting layer on a wide range of surfaces, followed by the formation of droplets of ILs.^38,51,55,72−78^ This behavior suggests that some ILs tend to spread evenly and form a continuous wetting layer on some substrates before droplet formation. The formation of continuous films has been observed for imidazolium-based ILs with long alkyl chains upon deposit on gold surfaces. The interaction between the polarizable gold atoms and the charged ions of the ILs results in the formation of “image dipoles”, which enhances the adsorption and ordering of the first ion pairs deposited on the surface. This initial favorable adsorption facilitates the improved spreading of the subsequent ML, especially for long-chain ILs, due to the dispersive interactions among the alkyl chains. These interactions contribute to the 2D growth process. Coalesced films have been experimentally observed, for instance, for [C_8_C_1_im][OTf] and [C_8_C_1_im][NTf_2_].^46,62,63^ On other surfaces, such as soda-lime glass, glass coated with conductive oxides, silver, graphene, nickel, as well as mica surfaces, the formation of droplets has been consistently observed for imidazolium-based ILs.^8,12,48,53,57,58,61−63,67^ The formation of larger droplets with enhanced wetting ability is further promoted when longer alkyl chain lengths are attached to the IL cation.^57,62,63,67^

Even in a vacuum environment, every surface inevitably acquires carbon contamination. In fact, in real-world scenarios, it is often challenging to completely eliminate carbon from surfaces. For practical applications, such as in electronics and optics, the goal is to minimize its presence and ensure that it does not negatively impact the intended function or performance of the surfaces or devices. Herein, our primary objective is to investigate how the presence of carbon films affects the surface morphology of IL films on different substrates. For this, we addressed the effect of carbon surface coating by examining the morphology of IL films on solid substrates previously coated with amorphous carbon (C). The ILs studied included short- and long-chain alkylimidazolium cations (C_2_C_1_im and C_8_C_1_im) paired with the anions bis(trifluoromethylsulfonyl)amide (NTf_2_) or triflate (OTf), and the experiments were conducted using identical deposition conditions and various surfaces.

## 2 Experimental Section

### 2.1 IL Samples

**Figure 1:**
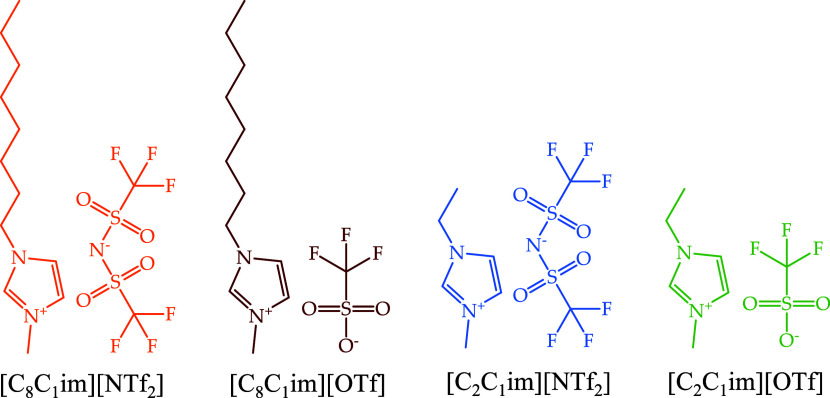
Molecular structures of the ILs under study and their respective acronyms: 1-methyl-3-octylimidazolium bis(trifluoromethylsulfonyl)amide, [C_8_C_1_im][NTf_2_]; 1-methyl-3-octylimidazolium triflate, [C_8_C_1_im][OTf]; 1-ethyl-3-methylimidazolium bis(trifluoromethylsulfonyl)amide, [C_2_C_1_im][NTf_2_]; and 1-ethyl-3-methylimidazolium triflate, [C_2_C_1_im][OTf].

1-Methyl-3-octylimidazolium bis(trifluoromethylsulfonyl)amide, [C_8_C_1_im][NTf_2_], 1-methyl-3-octylimidazolium triflate, [C_8_C_1_im][OTf], 1-ethyl-3-methylimidazolium bis(trifluoromethylsulfonyl)amide, [C_2_C_1_im][NTf_2_], and 1-ethyl-3-methylimidazolium triflate, [C_2_C_1_im][OTf], were commercially purchased from IoLiTec with a stated purity of 99%. The molecular structures of the studied ILs are listed in Figure 1.

Prior to initiating the deposition experiments, the ILs underwent a drying process under reduced pressure (<10 Pa), with continuous stirring for at least 48 h at *T* = 373 K. This procedure was used to minimize the presence of water or other volatile contents, reducing them to values in the ppm range. Inside the vacuum chamber, an additional cleaning experiment occurred at higher temperatures (*T* = 423 K). Generally, potential impurities, typically polymeric derivatives in the ppm range, exhibit a nonvolatile nature and thus do not interfere with the deposited films. Some relevant properties of the studied ILs are provided in Table S1 of the Supporting Information.

### 2.2 Substrates

The ILs were deposited on indium-tin-oxide-coated glass (ITO/glass), ITO/glass coated with silver (Ag/ITO/glass), and ITO/glass coated with gold (Au/ITO/glass). Additionally, the same substrates were coated with amorphous carbon (C/ITO/glass, C/Ag/ITO/glass, and C/Au/ITO/glass) to investigate its influence on the nucleation and growth of ILs. ITO/glass substrates with dimensions of 10 mm × 10 mm × 1.1 mm were purchased from Praezisions Glas & Optik GmbH. The ITO films had a thickness of approximately 180 nm, as indicated by the supplier. The substrates were washed with high-purity ethanol in an ultrasonic bath. Subsequently, the substrates were dried using pure argon and stored in a desiccator. To create metal surfaces (Ag/ITO and Au/ITO), the ITO substrates were coated with metallic films (Ag and Au) using the Cressington 108 Auto Sputter Coater instrument through direct current (DC) magnetron sputtering. Inside a vacuum chamber filled with pure argon, an electric field was applied to generate plasma.

The sputter deposition process utilized high-purity (>99.9%) metal targets, and the process was carried out with a discharge current of 40 mA. The thickness of the metallic films was monitored using a quartz crystal microbalance (QCM). The QCM was employed to measure the change in mass on the crystal surface as the metallic films were deposited, allowing for real-time monitoring of the film thickness. The deposition time was adjusted to achieve a desired thickness of approximately 100 nm. To coat the studied surfaces with carbon, carbon films with different thicknesses (10, 20, and 30 nm) were deposited using electron beam evaporation. The thickness of the carbon films was monitored in real-time using QCM. All the studied substrates were stored in a desiccator prior to the deposition experiments to maintain a controlled and dry environment. The duration of air exposure was minimized to reduce potential sample contamination.

### 2.3 Deposition of IL Films

Thin films of [C_8_C_1_im][NTf_2_], [C_8_C_1_im][OTf], [C_2_C_1_im][NTf_2_], and [C_2_C_1_im][OTf] were deposited by thermal evaporation, using a custom-built equipment developed in our laboratory.^79^ This system incorporates Knudsen effusion cells as evaporation sources, allowing for precise control of the mass flow rate. A comprehensive description of the methodology has been previously published,^79^ and the accompanying schemes can be found in Figures S1–S4 of the Supporting Information. The methodology employed ensures film deposition under highly reduced pressure conditions (*p* < 10^–4^ Pa). The utilization of a high-vacuum environment promotes the uniform movement of vapor particles, allowing them to directly reach the substrate surface and facilitate uniform deposition. To evaluate and control the film thickness, the Inficon model STM-2 QCM was employed. The mass flow rate (Φ) was determined using a modified form of the Knudsen equation, eq 1. The variables Φ_s_ and Φ_Kc_ represent the mass flow rates at the substrate surface and from the Knudsen cell orifice, respectively. The geometric factor (*g*) is dependent on the distance between the Knudsen cell and the substrate. *T* denotes the evaporation temperature, *p* represents the equilibrium vapor pressure, *w*_0_ is the transmission probability factor, *M* is the molar mass of the effused vapor, *m* stands for the mass of the vapor, *t* represents the effusion time, and *A*_0_ corresponds to the area of the Knudsen cell orifice.
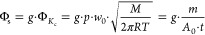
1

Each IL was deposited at varying amounts, ranging from 50 to 200 ML, onto substrates maintained at a constant temperature of *T* = (283.2 ± 0.2) K. The ML was used as a unit of coverage. The height (*h*) of 1 ML can be estimated using the empirical formula *h* = [*M*/(*N*_A_ρ)]^1/3^, where *M*, *N*_A_, and ρ represent the molar mass, Avogadro’s constant, and mass density, respectively. The values of these parameters for each IL are listed in Table S1. The estimated heights of 1 ML for each IL are as follows: [C_8_C_1_im][NTf_2_], *h* = 8.4 Å; [C_8_C_1_im][OTf], *h* = 7.8 Å; [C_2_C_1_im][NTf_2_], *h* = 7.5 Å; and [C_2_C_1_im][OTf], *h* = 6.8 Å. A constant deposition rate of Φ_s_ = (0.3 ± 0.1) Å/s was maintained for all depositions. The evaporation temperature and deposition time were carefully monitored to achieve the desired deposition rate and thickness. Detailed information regarding the deposition parameters for each IL, including the evaporation temperature, vapor pressure, and deposition rates, can be found in Tables S2 and S6 of the Supporting Information. The IL films were stored in a desiccator before their analysis. The morphological characterization was conducted 2–7 days after the deposition of the thin films, aiming to perform a time-dependent study of the film morphology.

### 2.4 XPS Analysis

X-ray photoelectron spectroscopy (XPS) was employed to characterize the ITO and Au substrates as well as the surface of the IL films deposited on both substrates. In addition, XPS was used to characterize the ITO surfaces coated with 10 and 20 nm amorphous carbon. The analysis was carried out at the Centro de Materiais da Universidade do Porto (CEMUP) using a Fi Kratos Axis Ultra HAS-VISION instrument. Monochromatic A1 Kα radiation (15 kV, 90 W) was used for the analysis, and surface areas measuring 300 μm × 700 μm were examined. The XPS survey spectrum of the substrates was obtained using a pass energy of 160 eV, a step size of 1.0 eV, and a dwell time of 200 ms. To subtract the background, the Shirley algorithm was applied before deconvolution of each spectrum into various components. For the IL film surfaces, high-resolution spectra were acquired for C 1s, O 1s, N 1s, F 1s, S 2p, In 3d, Sn 3d, and Au 4f using a pass energy of 40 eV and a step-size of 0.100 eV. All spectra were processed using CasaXPS software (version 2.3, Casa Software Ltd.).^80^ Relative sensitivity factors were obtained from the Kratos Library.

### 2.5 Morphological Analysis

Atomic force microscopy (AFM) was used to characterize the substrate morphology and quantitatively measure surface roughness. The AFM analysis was conducted at the CEMUP experimental facilities using the Multimode Nanoscope Atomic Force Microscope (Veeco Instruments Inc., USA) with a Bruker RTESP tip in tapping mode. Subsequently, the acquired images underwent processing and analysis using the Nanoscope AFM Analysis software.

The morphology of the IL samples was assessed using a high-resolution scanning electron microscope (FEI Quanta 400 FEG ESEM instrument) at CEMUP. Topographic images were acquired at various magnifications (500, 5000, 20,000, and 100,000×) to investigate the substrate surfaces as well as the micro/nanodroplets or coalesced films of the ILs. The micrographs were captured using a secondary electron (SE) detector positioned at a precise working distance of 10 mm and an accelerating voltage of 10 kV. The scanning electron microscopy (SEM) imaging parameters were meticulously regulated to mitigate the impact of the electron beam on the morphology of the IL films. Subsequently, specific micrographs were subjected to image processing analysis using ImageJ software^81^ to characterize the shape, surface coverage, and size distribution of the droplets.

## 3 Results and Discussion

### 3.1 Morphology and Roughness of the Substrates

**Figure 2:**
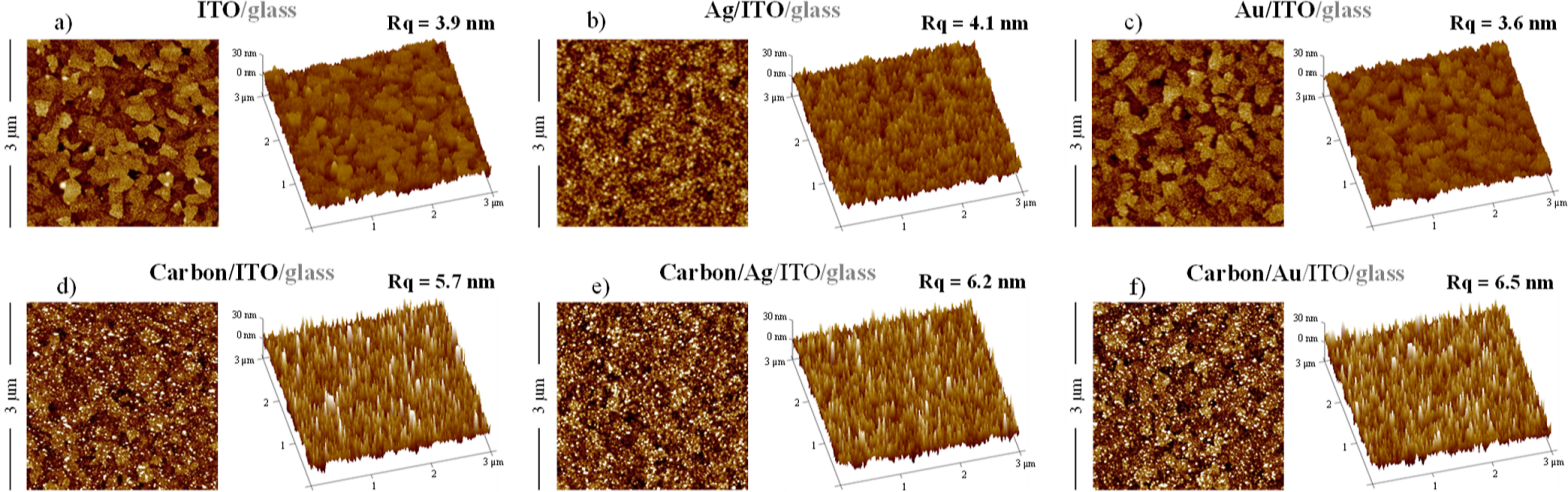
AFM images (3 μm × 3 μm), recorded in tapping mode, of the studied substrates: ITO/glass (a); Ag/ITO/glass (b); Au/ITO/glass (c); carbon/ITO/glass (d); carbon/Ag/ITO/glass (e); and carbon/Au/ITO/glass (f). The root-mean-square roughness (*R*_q_) for each surface is indicated alongside its respective image.

Figure 2 illustrates the morphology and roughness derived from AFM characterization of the surfaces under study, encompassing ITO, Ag/ITO, Au/ITO, C/ITO, C/Ag/ITO, and C/Au/ITO. Additionally, thin-film architectures and detailed SEM micrographs of the substrates are featured in Figure S5 of the Supporting Information.

According to the AFM images (3 μm × 3 μm), the root-mean-square roughness (*R*_q_) of ITO measured 3.9 nm, while for Ag/ITO, it was 4.1 nm, and for Au/ITO, it was 3.6 nm. Coating these surfaces with a thin layer of carbon resulted in increased *R*_q_ values: 5.7 nm for carbon/ITO, 6.2 nm for carbon/Ag, and 6.5 nm for carbon/Au.

### 3.2 Impact of 20 nm Carbon Coating on the Morphology of IL Films (50 to 200 ML) and the Effect of Cation Alkyl Chain Length

**Figure 3:**
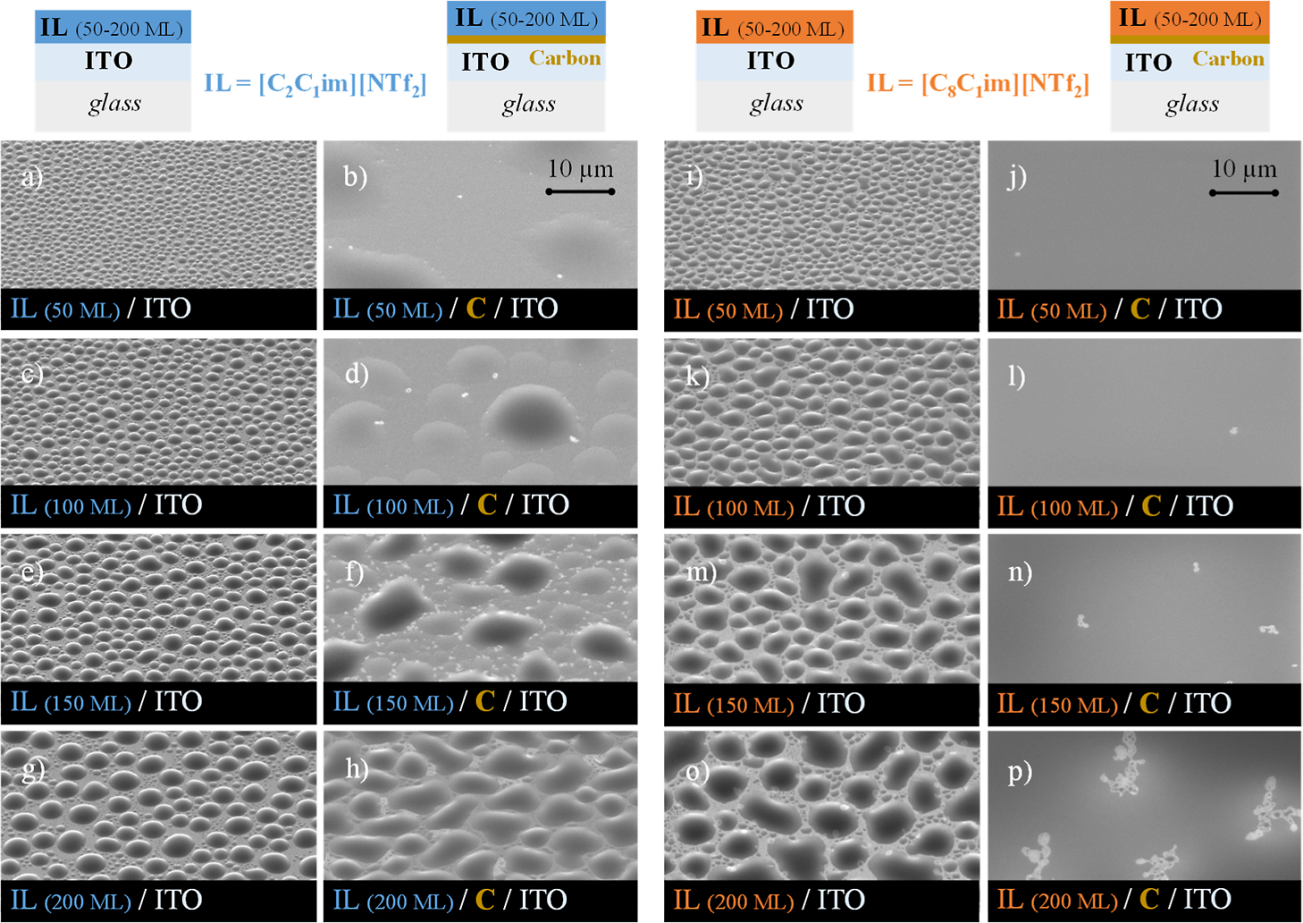
Thin-film architectures and detailed micrographs of thin films of [C_2_C_1_im][NTf_2_] (images a–h) and [C_8_C_1_im][NTf_2_] (images i–p) deposited on ITO/glass and carbon/ITO/glass surfaces (carbon thickness ≈20 nm). Each IL was deposited on both surfaces with varied thicknesses (50, 100, 150, and 200 ML). Micrographs were acquired at a lateral view of 45° with a magnification of 5000×, using a high-resolution scanning electron microscope and an SE detector.

Different amounts of [C_2_C_1_im][NTf_2_] and [C_8_C_1_im][NTf_2_] were deposited on ITO/glass and ITO/glass coated with carbon (20 nm of thickness). These ILs were chosen based on their differing cation alkyl chain lengths, which have been previously reported to affect the resulting film morphology.^57,62,63^Figure 2 indicates a change in the substrate morphology, accompanied by an increase in surface roughness (from 3.9 to 5.7 nm) following the deposition of the carbon film. Figure 3 displays the morphology of [C_2_C_1_im][NTf_2_] (Figure 3a–h) and [C_8_C_1_im][NTf_2_] (Figure 3i–p) deposited at 50 MLs (Figure 3a,b,i,j), 100 MLs (Figure 3c,d,k,l), 150 MLs (Figure 3e,f,m,n), and 200 MLs (Figure 3g,h,o,p) on both ITO/glass and C/ITO/glass substrates.

The experimental conditions for the deposition of these films are presented in Table S2. The successive increase in the amount of IL deposited (from 50 to 200 ML) on ITO/glass resulted in an increase in droplet size for both [C_2_C_1_im][NTf_2_] (micrographs depicted in the first column) and [C_8_C_1_im][NTf_2_] (micrographs depicted in the third column). However, the droplets were found to be larger for [C_8_C_1_im][NTf_2_] due to more intense coalescence mechanisms, in agreement with a previous study.^62^ The SEM micrographs were obtained from lateral views, which provided valuable data for inferring the shape and sphericity of the droplets. Analyzing the contact angle of microdroplets of ILs obtained by vapor deposition provides crucial information about the wetting behavior and surface interactions of the liquid on the substrate. According to Young’s equation (γ_sg_ = γ_sl_ + γ_lg_ cos θ_c_), effective liquid spreading on a solid substrate requires the liquid–gas interfacial tension (γ_lg_) to ideally be smaller than the difference between the solid–gas interfacial tension (γ_sg_) and solid–liquid interfacial tension (γ_sl_): γ_lg_ < γ_sg_ – γ_sl_.^82^ In the context of comparing [C_2_C_1_im][NTf_2_] and [C_8_C_1_im][NTf_2_] deposited on the same surface, the differentiation in the contact angle primarily arises from the magnitudes of γ_lg_ and γ_sl_. Lower values of both γ_lg_ and γ_sl_ contribute to a lower contact angle. The lower value of γ_lg_ for [C_8_C_1_im][NTf_2_] compared to that for [C_2_C_1_im][NTf_2_] (as shown in Table S1) contributes to the observed lower contact angles of the droplets obtained for this IL. Numerous factors affect the wetting behavior and contact angle of ILs, such as the type of cations and anions, substrate-IL affinity, substrate temperature, surface roughness, and deposition rate. Furthermore, recent studies have demonstrated that the contact angle greatly depends on the interface-induced solidified layer of ILs on the substrate.^83^ In a previous work, we investigated IL films on ITO and Ag surfaces using AFM and concluded that on both surfaces, the contact angles displayed values between 15 and 20°.^62^ In the study presented here, it can be inferred that the presence of the amorphous carbon layer induced the formation of the droplets with lower contact angles. This observation suggests improved wettability, as evidenced by the micrographs depicted in the second and fourth columns of Figure 3.

For larger thicknesses, the microdroplets of [C_8_C_1_im][NTf_2_] (Figure 3m,o) exhibit lower contact angles, whereas [C_2_C_1_im][NTf_2_] forms more spherical droplets (Figure 3e,g). Notably, the deposition of an IL on substrates coated with ≈20 nm of amorphous carbon film resulted in a significant change in the morphology of the IL film. In the case of [C_2_C_1_im][NTf_2_], it was observed that the lowest amount of IL deposition (50 ML) led to increased spreading and the formation of a continuous film (Figure 3b). This finding aligns with the work of Wang and Li, who reported that an imidazolium-based IL deposited on amorphous carbon exhibits a fairly smooth film for thinner nanofilms. The smooth film is attributed to the π–π parallel stacking between the imidazolium cation and the randomly distributed sp^2^ carbon on the amorphous carbon surface.^84^ According to Figure 3, with an increase in the amount of IL (50–200 ML), droplets formed on top of the carbon layer (Figure 3d,f,h). These droplets were larger compared to those deposited on ITO (without carbon). In the case of the highest IL amount (200 ML, Figure 3h), SEM images revealed the presence of an uncovered ITO surface: the carbon film seemed to have diffused along the surface (details can be observed in images h and p of Figure S6). This suggests partial miscibility between the IL and the carbon film, in line with the strong interaction between the imidazolium cation and the amorphous carbon surface.^84^

In the case of [C_8_C_1_im][NTf_2_], a continuous film of the IL is observed regardless of the amount deposited. When the ITO substrates are fully covered with carbon, there is complete wettability of [C_8_C_1_im][NTf_2_] as a result of the reduced values for both γ_lg_ and γ_sl_. Figure S6 presents zoomed-in views of the SEM images depicted in Figure 3. The high-resolution images reveal that with increasing amounts of C_2_C_1_im, droplet formation occurs, leading to a reduction in the continuous film. In the case of C_8_C_1_im, continuous film growth is consistently observed on top of the carbon layer. Nevertheless, some pinholes may form, indicating the presence of exposed ITO due to diffusion of the film. The uncovered areas of the substrate without carbon enable the deposition of new ion pairs directly on the ITO surface. This leads to the adsorption, nucleation, and formation of nanodroplets, as observed in image p of Figure S6.

The experiments clearly demonstrated distinct differences in the morphologies of IL films deposited on both ITO and C/ITO surfaces. Carbon coating on ITO creates a favorable surface for the IL to spread and adhere to, thereby enhancing its wetting characteristics on the substrate. ILs containing longer alkyl chains, such as [C_8_C_1_im][NTf_2_], demonstrate even better spreading behavior on carbon films. This enhanced behavior is attributed to the strong interactions between the imidazolium ring and the alkyl chain and the carbon surface. As a result, ILs with longer alkyl chains tend to form continuous films with improved coverage and reduced droplet formation when deposited on carbon-coated substrates.

There is no apparent influence of time on the morphology of the IL film, also on ITO surfaces coated with carbon as once the deposition is completed, the film’s morphology remains stable even after exposure to air for several days. The detailed results of the time-dependent study of the morphology and size distribution of IL droplets are presented in Figures S7–S10. Regardless of the resting time after deposition, the droplet size distribution obtained for the deposition of 50 MLs of [C_2_C_1_im][NTf_2_] (Figure S7) on ITO/glass shows a Gaussian size distribution, with the modal diameter (MD) approximately being 1 μm. When the deposition is performed on carbon-coated surfaces, the formation of larger, irregularly shaped droplets is observed. Their sizes were found to be similar regardless of the resting time after deposition. As a supplementary result, similar conclusions were obtained for [C_2_C_1_im][OTf] (Figure S9): MD near 1 μm on ITO/glass and larger droplets (MD above 2 μm) on the carbon film surface. Depositing a substantial amount of IL resulted in an enlargement of droplet size, which remained consistent irrespective of the duration between the deposition and subsequent examination (Figures S8 and S10).

### 3.3 Impact of 20 nm Carbon Coating on the Morphology of 100 ML IL Films: Investigating Substrate and Cation/Anion Variations

Aiming to examine the influence of carbon on other solid surfaces, IL films were deposited not only on carbon/ITO but also on carbon/Ag and carbon/Au substrates. The IL surface morphology was compared to that obtained on the same substrates without a carbon coating. It should be noted that metal films were produced on the ITO/glass surfaces. In order to infer the role of the carbon layer in the nucleation and growth of ILs, we chose to study surfaces with 100 ML as for this IL quantity, the ITO surface remains fully covered by carbon, in contrast to what is observed for 200 ML, where the partial miscibility between the IL and the carbon film would prevent differentiation of the effect. For the results shown in this section, 100 MLs of IL were deposited under identical experimental conditions (details in Table S3) on all surfaces, always with and without carbon.

**Figure 4:**
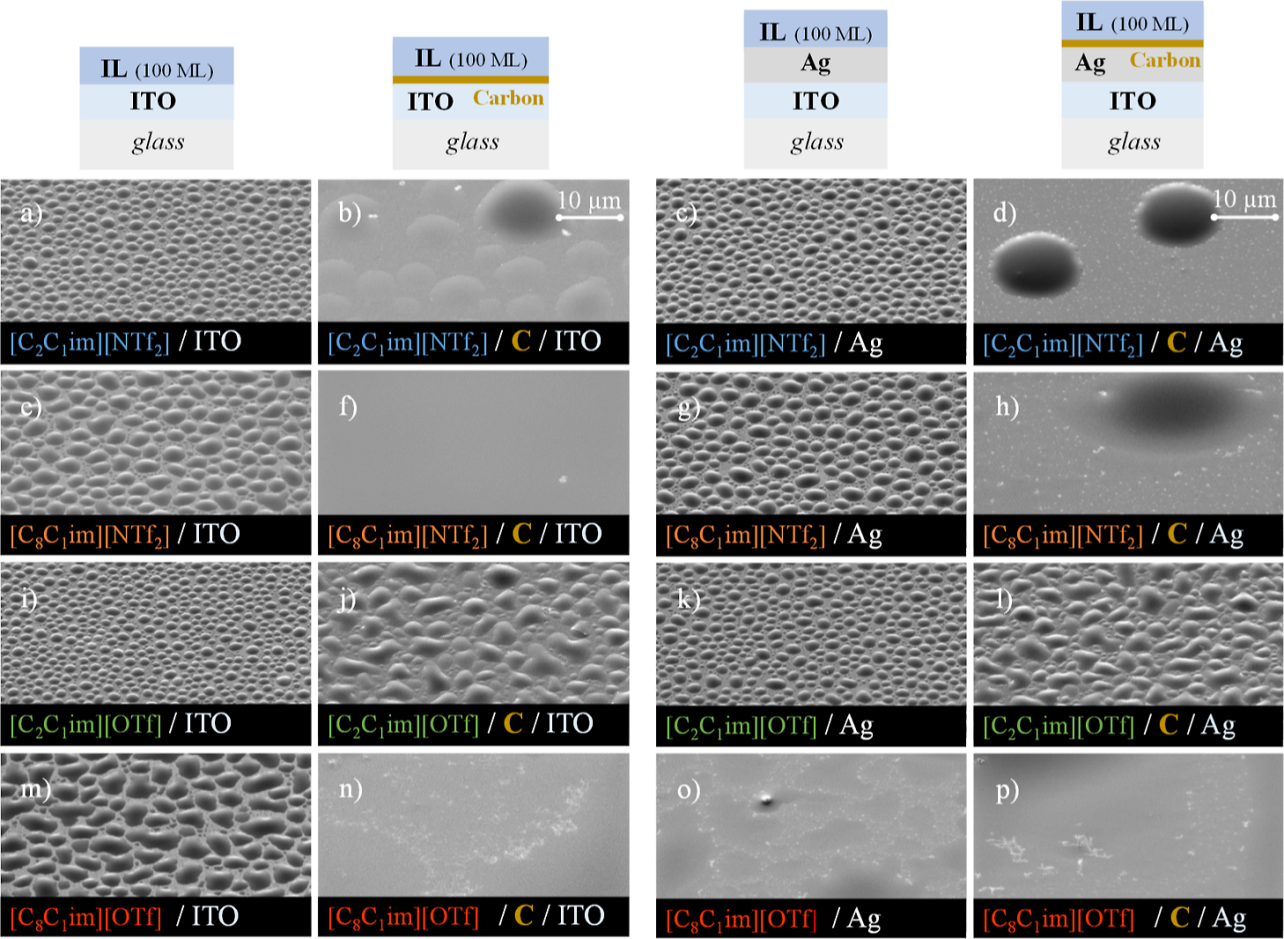
Thin-film architectures and detailed micrographs of thin films of [C_2_C_1_im][NTf_2_] (images a–d), [C_8_C_1_im][NTf_2_] (images e–h), [C_2_C_1_im][OTf] (images i–l), and [C_8_C_1_im][OTf] (images m–p) deposited on ITO/glass (first column), carbon/ITO/glass (second column), Ag/ITO/glass (third column), and carbon/Ag//ITO/glass (fourth column) surfaces (carbon thickness ≈20 nm). Each IL was deposited on both surfaces with a thickness of 100 ML. Micrographs were acquired at a lateral view of 45° with a magnification of 5000× using a high-resolution scanning electron microscope and an SE detector.

The experimental results are listed in Figures 4 and 6. Experimental data are presented for short-chain ([C_2_C_1_im][NTf_2_] and [C_2_C_1_im][OTf]) and long-chain ([C_8_C_1_im][NTf_2_] and [C_8_C_1_im][OTf]) alkylimidazolium-based ILs. Figure 4 depicts the morphology obtained for the four ILs deposited on ITO (the first column), C/ITO (the second column), Ag (the third column), and C/Ag (the fourth column). The morphology of the droplets of [C_2_C_1_im][NTf_2_] and [C_2_C_1_im][OTf] deposited on ITO (Figure 4a,i) and Ag/ITO surfaces (Figure 4c,k) is similar, in agreement with recent studies.^62,63,67^

**Figure 5:**
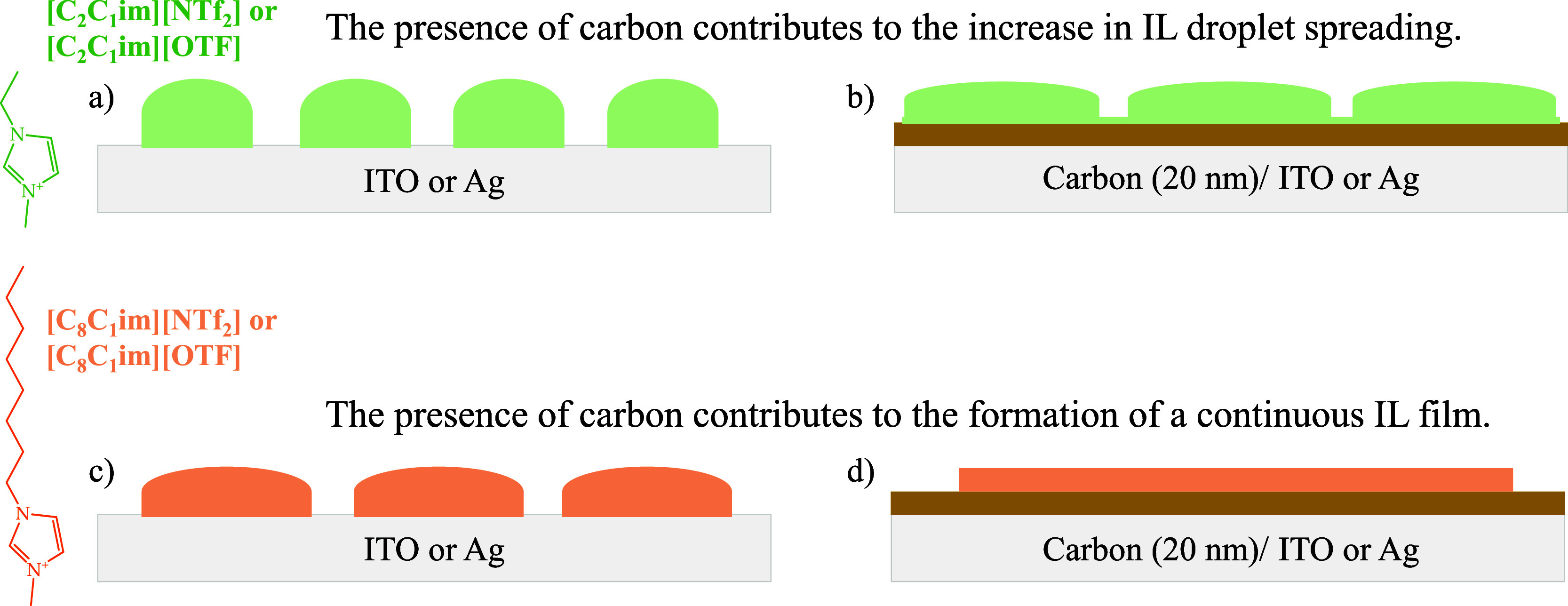
Schematic representation of the impact of carbon coating on the morphology and wetting behavior of [C_2_C_1_im][NTf_2_] and [C_2_C_1_im][OTf] (schemes a and b) as well as [C_8_C_1_im][NTf_2_] and [C_8_C_1_im][OTf] (schemes c and d) deposited on ITO or Ag surfaces.

**Figure 6:**
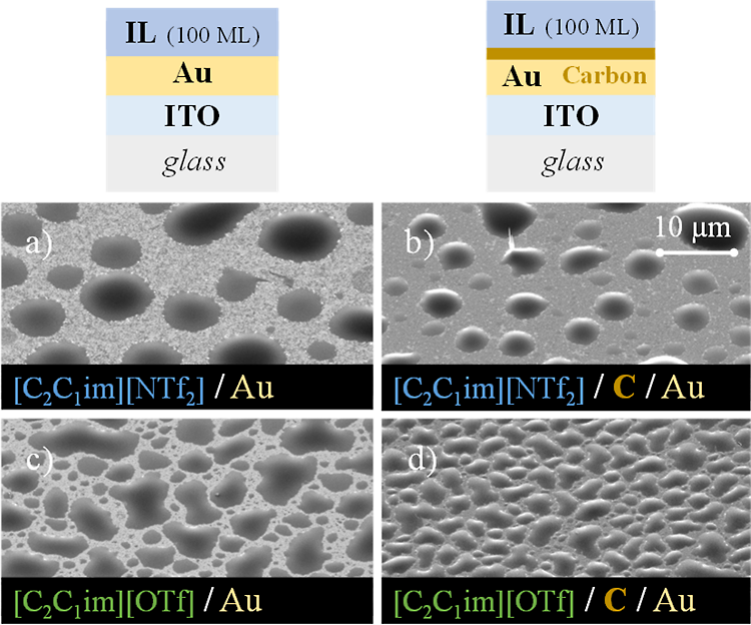
Thin-film architectures and detailed micrographs of thin films of [C_2_C_1_im][NTf_2_] (images a and b) and [C_2_C_1_im][OTf] (images c and d), deposited on Au/ITO/glass (first column) and carbon/Au/ITO/glass (second column) surfaces (carbon thickness ≈20 nm). Each IL was deposited on both surfaces with a thickness of 100 ML. Micrographs were acquired at a lateral view of 45° with a magnification of 5000× using a high-resolution scanning electron microscope and an SE detector.

There is no significant influence of the anion nature (NTf_2_ or OTf) or the substrate (ITO/glass or Ag/ITO/glass) on the morphology of the droplets formed by the thermal evaporation of C_2_C_1_im-based ILs. Larger droplets are formed for C_8_C_1_im-based ILs ([C_8_C_1_im][NTf_2_] and [C_8_C_1_im][OTf]) on both ITO and Ag/ITO surfaces (Figure 4e,g,m,o). However, the droplets of [C_8_C_1_im][OTf] obtained on Ag/ITO (Figure 4o) exhibit smaller contact angles compared to those with the other samples.

When the four ILs were deposited on the same substrates that had been previously covered with a thin film of carbon (20 nm), a significant change in the morphology was observed. Notably, for the same IL, similar morphologies are observed on C/ITO and C/Ag surfaces (comparing Figure 4b,d,f,h,j,l,n,p). Both [C_8_C_1_im][NTf_2_] and [C_8_C_1_im][OTf] exhibit complete spreading on C/ITO (Figure 4f,n) and C/Ag surfaces (Figure 4h,p). Formation of droplets is visible for [C_2_C_1_im][NTf_2_] (Figure 4b,d) and [C_2_C_1_im][OTf] (Figure 4j,l), but those formed on C/ITO and C/Ag are larger for [C_2_C_1_im][NTf_2_] (Figure 4b,d). Layer-by-layer growth followed by the formation of three-dimensional islands seems to have occurred for the short-chain ILs. Higher-magnification micrographs of the IL films deposited on C/ITO and C/Ag surfaces are presented in Figure S11. The morphological differentiation between the IL film deposited on “clean” ITO and Ag/ITO substrates, and on the same substrates previously covered with a thin film of carbon, is clear. 2D growth followed by the formation of very large droplets is observed for [C_2_C_1_im][NTf_2_] deposited on C/ITO and C/Ag (Figure 4b,d) whereas the deposition of [C_2_C_1_im][OTf] on substrates coated with carbon resulted in the formation of significantly large droplets (Figure 4j,l). Within the gaps between the droplets deposited on C/ITO, the underlying ITO is exposed, suggesting that the carbon film contracted, possibly being dissolved by the IL, leading to the growth of 3D islands, as typically observed for the ILs deposited directly on ITO/glass surfaces. The presence of smaller droplets between the larger ones supports the notion that these areas now have a minimal amount of carbon deposited. At odds, complete wettability is noted for both [C_8_C_1_im][NTf_2_] and [C_8_C_1_im][OTf] (Figure 4f,h,n,p). Figure 5 schematically represents the process of IL film growth on the surfaces of ITO and Ag, as well as on the same surfaces coated with amorphous carbon. The results obtained here suggest that the presence of a carbon film significantly enhances the wettability of the IL films. The use of thinner IL films demonstrates the potential for various interfacial applications in electronic devices. The possibility of employing small quantities of carbon as a strategy to spread an IL film holds promise for enhancing the effectiveness of these films as solvents, particularly in vacuum conditions, such as in the crystallization of organic semiconductors.^8,12,60^

In recent studies, it was found that long-chain ILs exhibit complete spreading onto Au surfaces, resulting in the growth of the IL film layer by layer.^62,63^ This suggests that identical morphologies would likely be obtained on both Au and C/Au surfaces with a homogeneous and compact IL film covering the entire surface. Differentiation is expected for the short-chain ILs. Consequently, the deposition of [C_2_C_1_im][NTf_2_] and [C_2_C_1_im][OTf] on both Au and C/Au substrates was investigated in this study. Figure 6 depicts the morphology obtained for these ILs deposited on Au (the first column) and C/Au (the second column). The experimental results show the formation of large droplets on both surfaces. Despite some differences, the morphology of [C_2_C_1_im][NTf_2_] did not undergo significant changes when Au was coated with C. This contrasts with the pronounced differentiation observed in the case of the ITO and Ag substrates.

Upon examination of the substrate surfaces (Figure 2), it becomes evident that both the carbon-coated surfaces exhibit similar morphologies, irrespective of the underlying substrate (ITO, Ag, or Au). The thickness of the carbon coating layer would be expected to be relatively substantial to mitigate the direct interaction of the ILs with the substrates. Additionally, in both cases, there is an observed increase in roughness when the surfaces are coated with amorphous carbon. Based on the results obtained with C/ITO and C/Ag, one would expect a higher spreading behavior on C/Au compared to that on clean Au. However, experimental results did not confirm this expectation. It is well-known that Au possesses unique properties and exhibits strong interactions with ILs.^62,63^ Au, with its distinct surface energy and chemical properties, may exhibit variations in surface charge and polarizability when compared to Ag and ITO. These differences can influence the intermolecular forces between the substrate, carbon coating, and IL. Furthermore, Au is recognized for its electrochemical stability, which could impact the wetting behavior. Interestingly, the morphology of [C_2_C_1_im][OTf] on C/Au, C/Ag, and C/ITO is quite similar. The droplets appear heterogeneous and less spherical as compared to those observed for [C_2_C_1_im][NTf_2_] on Au and C/Au. Higher-magnification micrographs (Figure S12) suggest that for [C_2_C_1_im][NTf_2_], a large amount of the Au surface is coated with IL microstructures exhibiting reduced contact angles. Layer-by-layer growth followed by the formation of 3D islands seems to have occurred. On the other hand, for [C_2_C_1_im][OTf], it appears that the areas between the large droplets remain uncovered with IL. These droplets exhibit lower apparent contact angles on both Au and C/Au surfaces in comparison to those of [C_2_C_1_im][NTf_2_]. The quartz crystals (QCs) used for monitoring the deposition rate are gold-coated. To conduct an additional enlightening experiment, a carbon film was applied to the Au/QC surface, and the morphology of the IL films deposited under identical conditions on Au/QC and C/Au/QC surfaces was evaluated. The results are presented in Table S4 and Figure S13. According to the data, most of [C_2_C_1_im][NTf_2_] has been incorporated into the confined spaces of the QC, regardless of the material used to coat the QC surface. However, when the deposition was directly carried out on the Au/QC, the presence of an IL was clearly observed on the surface. On the QC coated with C, there is no visible presence of the IL at the surface. The carbon film might have diffused along with the ion pairs into the confined spaces of the QC. Alternatively, there may be an ultrathin film of the IL that is undetectable by SEM.

### 3.4 XPS Characterization of the Substrates and the IL Films

According to our experimental results, the carbon film has a significant impact on the morphology of the thermally evaporated ILs. In theory, the solid substrates studied (ITO, Ag, and Au), which were not coated with a carbon film, might not be completely devoid of carbon contamination. The presence of carbon contamination can be attributed to a variety of factors, including exposure to air, handling, and manufacturing processes. In numerous environments, carbon-based compounds are abundant and have the potential to accumulate gradually on surfaces. This contamination can manifest as either carbon-containing particles or adsorbed carbonaceous substances. Even under vacuum conditions, any surface is inevitably contaminated to some extent by different carbon species. The influence of surface carbon on IL wetting has indeed been investigated by several surface science groups.^53,85−87^ A study on the physical vapor deposition (PVD) of imidazolium-based ILs using mica surfaces as the substrate has shown that on a clean mica surface (without any detectable carbon species by XPS), a 3D growth of the IL film occurred. However, on a fully carbon-covered surface, the IL film initially formed a complete 2D wetting layer, followed by a 3D growth.^53^ In the present study, we obtained similar conclusions regarding the influence of surface carbon on the IL wetting for both ITO and Ag surfaces. It is important to mention that the magnitude of the changes induced by carbon in the morphology and wetting behavior of ILs highly depends on the length of the alkyl chains attached to the imidazolium cation, as well as on the type of substrate.

**Figure 7:**
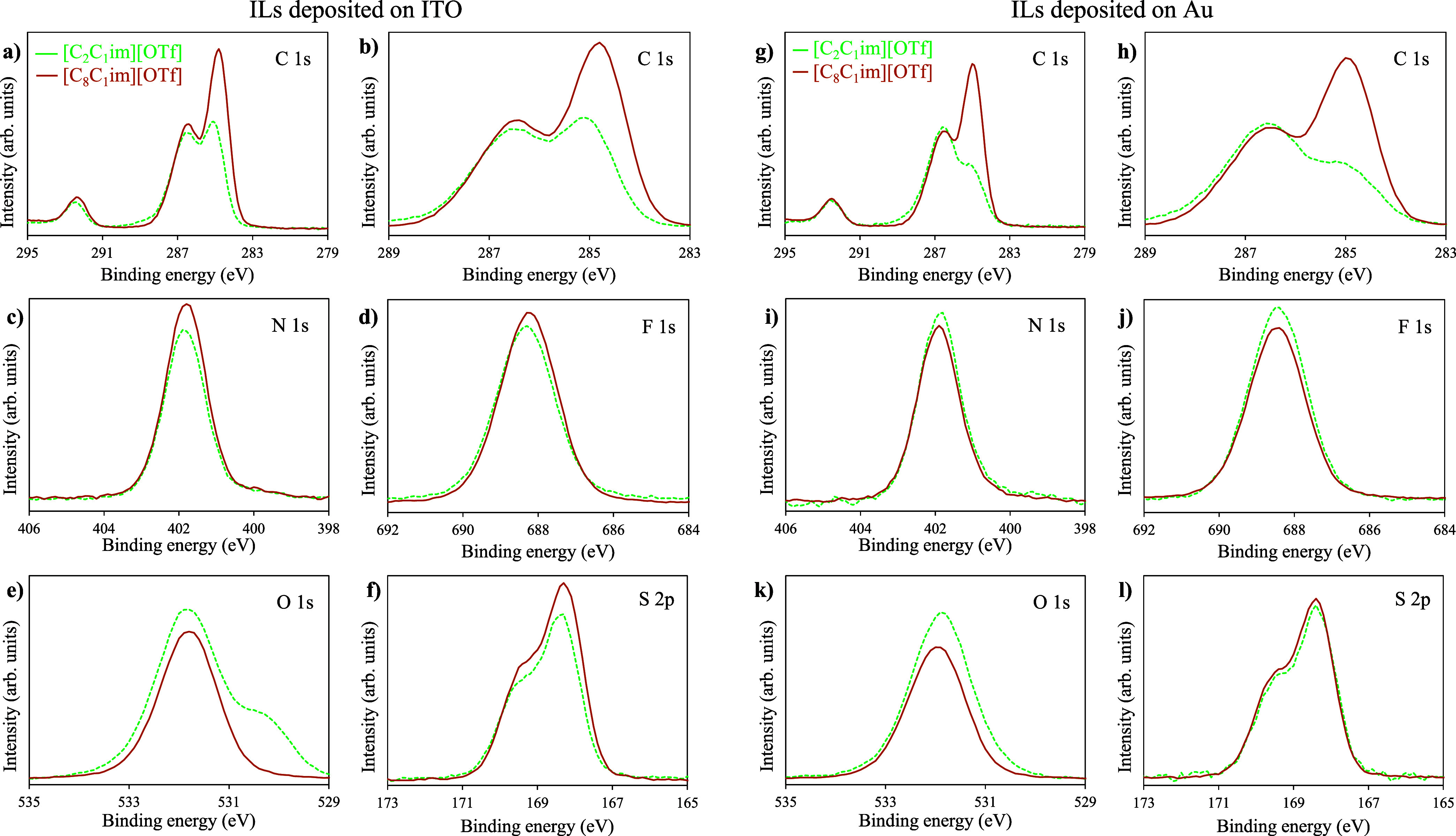
High-resolution XPS spectra of [C_2_C_1_im][OTf] (dashed lines) and [C_8_C_1_im][OTf] (solid lines) film surfaces deposited on ITO (spectra a–f) and Au (spectra g–l). The XPS spectra were acquired for C 1s (graphs a–h), N 1s (graphs c and i), F 1s (graphs d and j), O 1s (graphs e and k), and S 2p (graphs f and l).

XPS analysis was used here to assess the extent of carbon contamination in the ITO/glass and Au/ITO/glass substrates. Prior to the deposition experiments, these surfaces were thoroughly cleaned and stored appropriately. It is important to note that XPS spectra can be influenced by the presence of an adventitious carbon contamination layer on the surface, given the high sensitivity of the technique. Consequently, the obtained results may be influenced by incident carbon contamination originating from the unavoidable handling the substrates and their exposure to air. Additionally, during the deposition process, there is a potential for additional accumulation of contamination on the surface due to IL evaporation under moderate vacuum conditions. XPS survey spectra for the ITO and Au substrates are presented in Figures S14 and S15. Two different areas measuring 300 μm × 700 μm were analyzed for each surface, and consistent results were obtained. Based on the obtained relative atomic percentages, carbon amounts of (45 ± 1) and (25 ± 1)% were determined for the ITO and Au surfaces, respectively. These results highlight the higher susceptibility of the ITO surfaces to carbon contamination compared to that of the Au surfaces. In principle, any sample can be contaminated with adventitious carbon with a thickness of approximately 1 nm, even with a short exposure to the atmosphere.^88^ This level of contamination can indeed impact the adsorption of ILs on solid surfaces during the initial stages of film deposition. High-resolution XPS data for the short-chain IL, [C_2_C_1_im][OTf], and the long-chain IL, [C_8_C_1_im][OTf], are presented in Figure 7. High-resolution XPS spectra were acquired for C 1s, N 1s, F 1s, O 1s, and S 2p on the IL film surface on ITO (Figure 7a–f) and Au (Figure 7g–l). XPS survey spectra are shown in Figures S16–S19. For both ILs, the C 1s spectrum (Figure 7a,b,g,h) reveals distinct peaks at binding energies (BEs) that correspond to different carbon atom environments within the IL. The observed peaks are associated with specific carbon bonds, including C–C and C=C bonds (BE ≈ 285 eV), C–N and C–O bonds (BE ≈ 286 eV), and C–F bonds (BE ≈ 292 eV). These peaks provide valuable insights into the chemical bonding of carbon atoms within the cation (C–C, C = C, C–N, and C–O bonds) and the carbon atoms within the anion (C–F bonds) of the IL. For both ILs, [C_2_C_1_im][OTf] and [C_8_C_1_im][OTf], observed on both ITO and Au surfaces, there is a similarity in the intensity of the peaks corresponding to the C–N, C–O, and C–F components. However, there is a notable difference in the intensity of the C–C component, which is significantly higher for [C_8_C_1_im][OTf] (Figure 7b,h). This disparity in intensity can be attributed to the increasing length of the alkyl chain present in [C_8_C_1_im][OTf]. As the chain length increases, it contributes to a higher proportion of C–C bonds within the IL structure. This result indicates the influence of the alkyl chain length on the chemical composition and bonding characteristics of the IL film, as observed in the XPS spectra. Interestingly, for the [C_2_C_1_im][OTf] samples, the intensity of the C–C component is higher on the ITO surface (Figure 7b) compared to that observed on Au (Figure 7h). This phenomenon is likely a result of the presence of larger contamination by adventitious carbon on the ITO surface. SEM images reveal the formation of numerous droplets of the IL on the ITO surface, causing a significant portion (approximately 50%) of the substrate to remain uncovered by the IL. When a substrate is partially coated by ILs, typically in the form of droplets, the XPS signal arises from both the ITO surface and the IL/ITO surface. Consequently, the obtained results regarding the C 1s components also include information about the substrate, and the carbon contamination appears to contribute to the overall XPS spectrum. Since the carbon contamination is lower on the Au surfaces, the intensity of the peak associated with C–C components is correspondingly lower. The In 3d, Sn 3d, and Au 4f scan spectra are presented in Figure S20. In the case of the short-chain IL, C_2_C_1_im, the presence of peaks related to the substrate is evident for both the ITO and Au surfaces. However, for the long-chain IL, C_8_C_1_im, only a slight intensity of the peaks related to the substrate was found. This analysis is consistent with the observed better wetting behavior and higher surface coverage achieved with C_8_C_1_im. The N 1s spectra (Figure 7c,i), F 1s spectra (Figure 7d,j), the O 1s spectra (Figure 7e,k), and the S 2p spectra (Figure 7f,l) demonstrate the presence of expected peaks associated with nitrogen in the imidazolium cation, as well as fluorine, oxygen, and sulfur of the triflate anion. The analysis of N/F and N/S ratios provides valuable insights into predicting the cation-to-anion ratio on the film surface. Experimental values of N/F and N/S ratios were obtained for the studied ILs deposited on both ITO and Au surfaces. Specifically, for [C_2_C_1_im][OTf] deposited on ITO, the measured N/F ratio was determined to be 1.6:3, which deviated slightly from the expected ratio of 2:3.

Additionally, the measured N/S ratio was found to be 1.9:1, whereas the expected ratio would be 2:1. It is worth noting that the expected ratios are based on a cation-to-anion ratio of 1:1. For [C_2_C_1_im][OTf] deposited on Au, the experimental values yielded an N/F ratio of 1.6:3, which was consistent with the ratio observed on ITO. However, a differentiation was observed in the N/S ratio, which was determined to be 2.1:1. In the case of [C_8_C_1_im][OTf], N/S ratios of 1.8:1 and 1.9:1 were observed on ITO and Au surfaces, respectively. Additionally, N/F ratios of 1.7:3 were consistently observed on both surfaces. Details of the analysis are presented in Table S7. These experimental results indicate that the cation-to-anion ratios in the film surfaces of both [C_2_C_1_im][OTf] and [C_8_C_1_im][OTf] samples remain relatively consistent between ITO and Au surfaces in terms of the N/F ratio. However, there is a slight variation in the N/S ratio, with larger values being observed for the Au surface. Overall, these findings confirm the elemental composition of the IL surface. Additionally, the presence of adventitious carbon contamination was observed on both substrates, with higher levels detected on the ITO surface.

The effective thickness of adventitious carbon is significantly lower compared to that of the carbon layers here studied. Hence, we conducted extra XPS experiments to characterize the ITO surfaces coated with approximately 10 and 20 nm of carbon. XPS survey spectra for carbon (10 nm)/ITO and carbon (20 nm)/ITO substrates are presented in Figures S21 and S22. Our findings strongly suggest that the carbon film effectively covers the entire ITO surface. For the 20 nm sample, the signal from the ITO is almost imperceptible, strongly indicating that the carbon coating completely isolates the ITO from direct contact with the ILs. In the case of the 10 nm sample, the signal from ITO is just barely perceptible, consistent with the thickness of the carbon layer and XPS information.

### 3.5 Impact of Carbon Coating (10 to 30 nm) on the Morphology of 100 ML IL Films and the Effect of Cation Alkyl Chain Length

**Figure 8:**
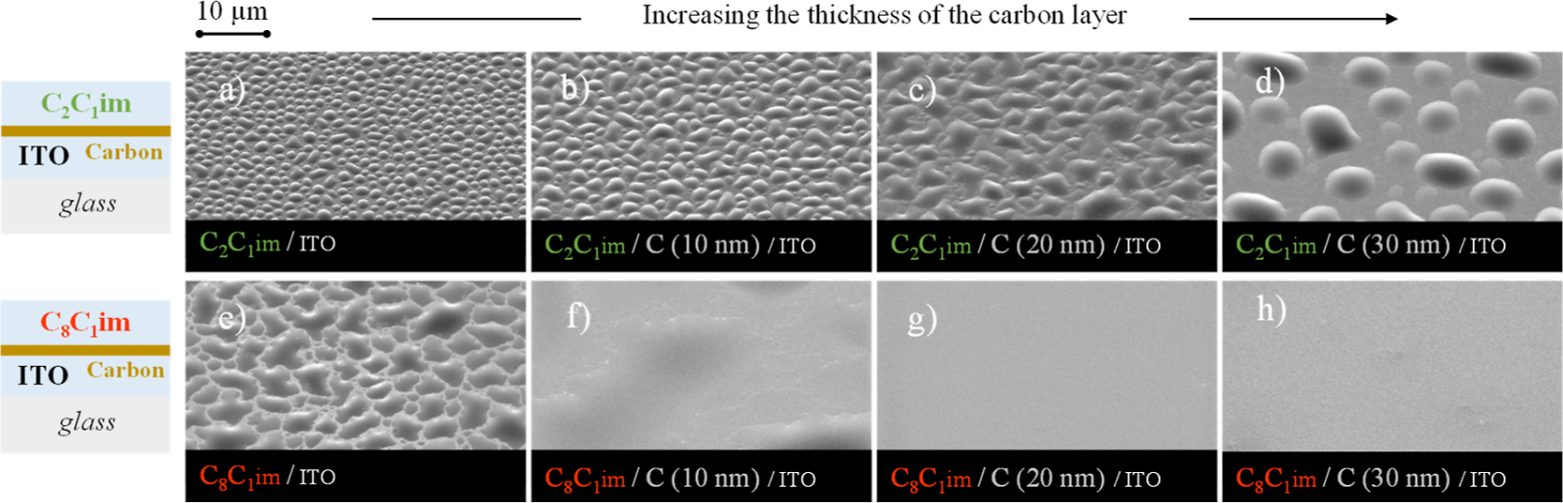
Thin-film architectures and detailed micrographs of [C_2_C_1_im][OTf] (images a–d) and [C_8_C_1_im][OTf] (images e–h) films (100 ML) deposited on ITO/glass (a, e) and carbon/ITO/glass (b–d, f–h). Each IL was deposited on ITO surfaces coated with varying amounts of carbon: 0 nm (a,e); 10 nm (b,f); 20 nm (c,g); 30 nm (d,h). Micrographs were acquired at a lateral view of 45° with magnification of a 5000× using a high-resolution scanning electron microscope and an SE detector.

The XPS analysis confirmed the presence of adventitious carbon on the substrates used. However, the estimated thickness of this carbon layer is less than 2 nm, which is significantly lower compared to that of the coated carbon surfaces studied earlier (approximately 20 nm). Furthermore, the distribution of the adventitious carbon layer across the entire substrate surface is expected to be nonuniform. On the other hand, considering the greater morphological differentiation induced by a carbon layer, the presence of adventitious carbon on the substrate surface can be relevant for the initial stages of nucleation and growth of IL films. In this context, to assess the impact of carbon thickness on the morphology of the IL films, [C_2_C_1_im][OTf] and [C_8_C_1_im][OTf] were deposited at 100 ML on carbon-coated ITO surfaces with varying thicknesses of the carbon layer, specifically 10, 20, and 30 nm. We have selected a short-chain IL (C_2_C_1_im) and a long-chain IL (C_8_C_1_im) because the results presented above have demonstrated noticeable differentiation in the morphologies of their films formed on C/ITO surfaces. The OTf anion was chosen because we observed a striking similarity in the film morphology of [C_2_C_1_im][OTf] on all carbon-coated surfaces. This choice allows us to focus on inferring the effect of the carbon thickness. Although the investigation of IL deposition on thinner carbon-coated surfaces would be of interest, limitations in experimental precision prevented the extension to thinner coatings as we cannot guarantee the quality of the carbon coating with a thickness of less than 10 nm. The obtained results are presented in Figure 8. The deposition conditions were identical for both ILs (Table S5), allowing for a direct comparison of the effects of the carbon thickness on the film morphology. The SEM images at the top of Figure 8 depict the deposition of [C_2_C_1_im][OTf], while those at the bottom show the deposition of [C_8_C_1_im][OTf]. SEM images with even higher resolutions are presented in Figure S23. From left to right in the images, the thickness of the carbon layer progressively increases to 30 nm.

Figure 8a,e displays the morphologies of the [C_2_C_1_im][OTf] and [C_8_C_1_im][OTf] films deposited on ITO substrates that are not covered with carbon in a controlled way, although it is important to consider the possibility of the presence of adventitious carbon. In the case of the short-chain IL, the presence of droplets is evident, with their size progressively increasing when the ITO substrate is coated with a thicker carbon film (Figure 8a–d). The [C_2_C_1_im][OTf] films initially appear to undergo a 2D growth mode, followed by the formation of 3D islands. As the thickness of the carbon layer increases, it can introduce alterations in surface energy and surface roughness. These changes, in turn, can influence the spreading dynamics of the ion pairs, potentially resulting in the formation of larger droplets of [C_2_C_1_im][OTf]. When considering the long-chain IL, [C_8_C_1_im][OTf], a significant change in the morphology is observed on the ITO substrate when coated with carbon. This change is evident in Figure 8e–h, already clear in the thinner carbon film (Figure 8f). In contrast to what was observed with C_2_C_1_im, the presence of a carbon film induces a 2D growth of the [C_8_C_1_im][OTf] film without the subsequent formation of droplets. Interestingly, as the thickness of the carbon layer increases, the formation of a compact and coalesced IL film increases. A comparison among Figure 8a,b,e,f reveals that [C_8_C_1_im][OTf] exhibits more significant morphological changes when the ITO substrate is coated with carbon as compared to those in [C_2_C_1_im][OTf]. The carbon film may serve as a template, creating a favorable surface for spreading and arrangement of C_8_C_1_im cations. This is facilitated by the longer alkyl chains, which exhibit enhanced affinity and stronger interactions with the carbon film. Due to their higher degree of flexibility, the alkyl chains can readily conform to the surface, promoting improved spreading and arrangement of the cations on the carbon-coated substrate. Figure 8e depicts the formation of droplets rather than continuous films (as observed in Figure 8f–h). Although there may be adventitious carbon present on the ITO surface, it did not significantly alter the morphology compared to the surfaces fully covered by carbon. However, it is worth noting that the irregular shapes of the droplets observed in Figure 8e, arising from the coalescence of neighboring droplets, could also be influenced by the increased wettability of the surface induced by the adventitious carbon. Consequently, it is crucial to minimize carbon contamination to ensure that the film morphology is solely governed by the intrinsic properties of the IL, the surface roughness, and the chemical composition of the clean substrate.

### 3.6 Impact of Cation Alkyl Chain Length on the Morphology of 100 ML IL Films at the ITO–Carbon Interface

**Figure 9:**
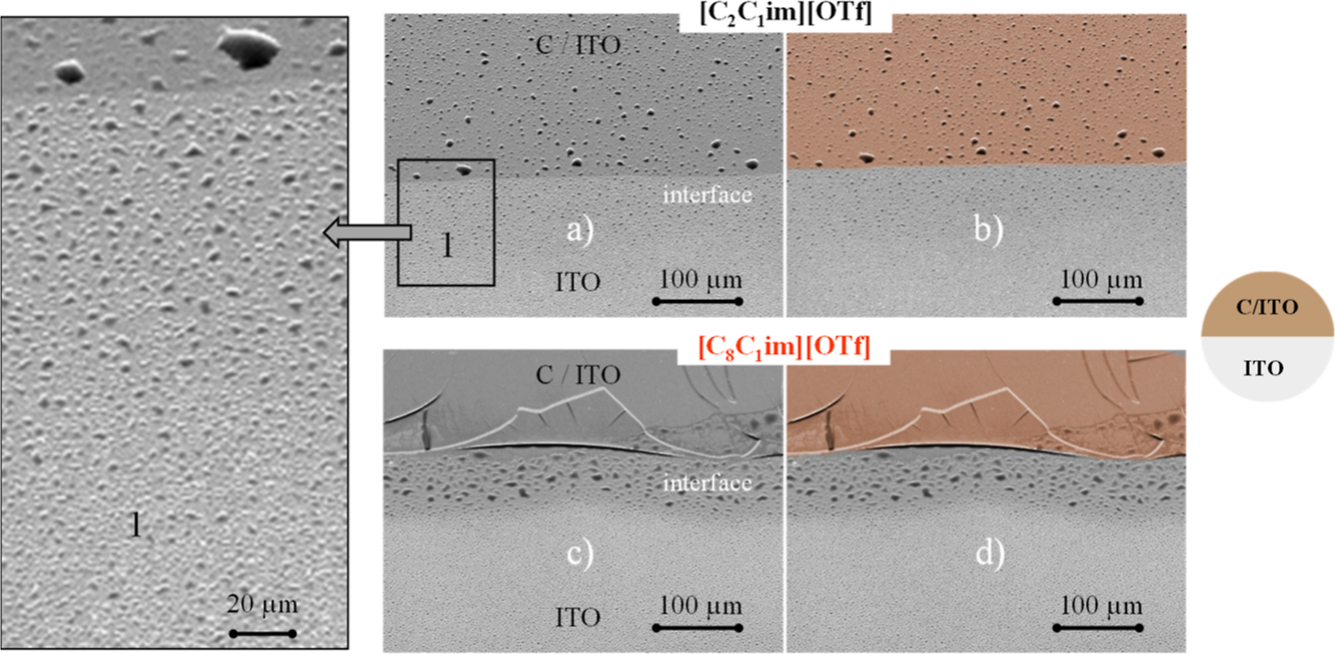
Micrographs of [C_2_C_1_im][OTf] (a,b) and [C_8_C_1_im][OTf] (c,d) simultaneously deposited on ITO/glass and carbon/ITO/glass. Images b and d are colored versions of SEM micrographs a and c, respectively, highlighting the carbon-covered portion of the substrate. Micrographs were acquired at a lateral view of 45° with a magnification of 500× using a high-resolution scanning electron microscope and an SE detector. A zoomed-in image of the interface is presented in image 1.

Figure 9 illustrates the SEM micrographs that depict the morphology of IL films (100 ML) on the ITO and carbon-coated ITO surfaces, including the region near the interface of both surfaces.

These images provide evidence of the greater affinity of the long-chain IL toward the carbon surface. In the case of [C_2_C_1_im][OTf] (Figure 9a,b), the formation of larger droplets is evident on the carbon-coated surface, consistent with the previously presented results for this IL. Near the interface, more pronounced droplet coalescence mechanisms are observed, indicative of enhanced surface diffusion—on the ITO surface, larger droplets prevail in the proximity of the interface, while smaller droplets are visible in regions further away from the interface. This observation is evident in the zoomed image of the interface (image 1 of Figure 9). For [C_8_C_1_im][OTf], droplets are formed on the ITO surface, while a coalesced film is observed on the carbon surface. At the interface, the adhesion between the carbon film and the ITO surface weakens, causing the carbon film to lose its adherence to the ITO surface. Interestingly, larger droplets can be observed on the ITO surface that was previously coated with the carbon film. This phenomenon is a result of the diffusion of ion pairs onto the ITO surface and beneath the carbon film, where it is likely that there are adsorbed carbon atoms, which contribute to enhanced spreading and subsequently intensify droplet coalescence. This observation supports the experimental suggestion that adventitious carbon cannot be overlooked in analyzing the wetting behavior of ILs, particularly those with long alkyl chains.

## 4 Conclusions

This study examined the effects of a carbon coating on the morphology of IL films with different thicknesses deposited on different solid substrates, with and without carbon coating, also at different thicknesses. The ILs varied in the cation alkyl side chain length and anion type. The thin films were created using vacuum thermal evaporation on ITO, Ag, and Au substrates, both with and without an amorphous carbon coating. The presence of a carbon coating on the ITO and Ag surfaces led to significant morphological changes in the IL films. These carbon-coated substrates produced films with increased wettability, particularly for long-chain ILs. The interaction between the alkyl chains and the carbon surface resulted in a layer-by-layer growth pattern. Conversely, no substantial alterations in the morphology of the IL films were observed when Au substrates were coated with carbon compared with those of clean Au surfaces.

An increase in the carbon thickness further elucidated the induced morphological changes in the IL films. The experimental data strongly suggested that higher levels of adventitious carbon contamination can potentially impact the adsorption of the first MLs of IL, even on surfaces assumed to be free of carbon coating. This influence was particularly significant for long-chain ILs. Higher levels of carbon at the substrate surface and lower quantities of IL deposition led to more pronounced morphological changes in the IL films.

In summary, the experimental results reported herein show that the morphological changes in the IL film are more pronounced when larger amounts of carbon are present at the substrate surface as well as when lower quantities of IL are deposited. Consequently, studies on the nanoscale need to be carried out with well-defined solid substrates as even very small amounts of carbon might influence the nucleation and growth of the first ML. At a mesoscopic level, carbon-induced changes can also be relevant for the wetting behavior, morphology, and growth tendency of thin IL films, especially for long-chain ILs, as highlighted in this work.

In terms of the scientific and technological significance of investigating thick layers of amorphous carbon, our findings indicate that the presence of a carbon film indeed improves the wettability of the IL films. The application of an IL coating in the form of a continuous film, rather than droplets of various shapes and sizes, indeed holds high potential for applications in molecular electronics as these layers, particularly, could serve as valuable interfacial materials.

## Supporting Information Available

Vapor deposition methodology, molecular structure of the ILs studied, thin-film architectures, and high-resolution micrographs of IL films deposited on different solid substrates used both with and without additional carbon coating, XPS spectra of the substrates and the IL film surface, physicochemical properties of ILs, experimental conditions for the physical vapor deposition of each IL, and experimental data derived from the XPS analysis (PDF)
