# Supplementary material for: Carbon-Induced Changes in the Morphology and Wetting Behavior of Ionic Liquids on the Mesoscale
Source: Langmuir. 2024 Feb 12;40(7):3949–61. doi: 10.1021/acs.langmuir.4c00102 (PMC10883047; doi:10.1021/acs.langmuir.4c00102)
Supplement: Supplementary file 1 — la4c00102_si_001.pdf [file la4c00102_si_001.pdf]

Supporting Information (SI)

Carbon-Induced Changes in the Morphology and Wetting Behavior of Ionic Liquids on the Mesoscale

*Rita M. Carvalho, Luís M. N. B. F. Santos, Margarida Bastos, and José C. S. Costa**

CIQUP, Institute of Molecular Sciences (IMS), Department of Chemistry and Biochemistry, Faculty of Science, University of Porto, Rua do Campo Alegre s/n, P4169-007 Porto, Portugal.

Figures and schemes depicting the vapor deposition methodology (Figures S1-S4), molecular structure of the ionic liquids studied, thin-film architectures, and high-resolution micrographs of IL films deposited on different solid substrates used both with and without additional carbon coating (Figures S5-S13 and S23), XPS spectra of the substrates and the IL film surface (Figures S14-S22), as well as tables reporting physicochemical properties of ILs (Table S1), experimental conditions for the physical vapor deposition of each ionic liquid (Tables S2-S6) and experimental data derived from the XPS analysis (Table S7), are presented.

*Corresponding author

jose.costa@fc.up.pt

List of Figures and Tables

Figure S1. Schematic representation of the vacuum thermal evaporation methodology. S4

Figure S2. Schematic representation of the ovens of the ThinFilmVD apparatus. S4

Figure S3. Schematic representation and images of the substrate support system. S5

Figure S4. Schematic illustration of the typical mechanisms of nucleation and growth of ionic liquid films obtained by vapor deposition. S5

Figure S5. Molecular structures of the ILs under study, thin-film architectures, and detailed micrographs of the substrates. S6

Figure S6. Thin-film architectures and detailed micrographs of thin films of $[C_2C_1im][NTf_2]$ and $[C_8C_1im][NTf_2]$ deposited at different amounts on ITO/glass and carbon/ITO/glass surfaces. S7

Figure S7. Thin-film architectures and detailed micrographs of $[C_2C_1im][NTf_2]$ films (50 ML) deposited on ITO/glass and carbon/ITO/glass surfaces. The morphology of the samples was assessed after 7 days, 4 days, and 2 days of film deposition. S8

Figure S8. Thin-film architectures and detailed micrographs of $[C_2C_1im][NTf_2]$ films (150 ML) deposited on ITO/glass and carbon/ITO/glass surfaces. The morphology of the samples was assessed after 7 days, 4 days, and 2 days of film deposition. S9

Figure S9. Thin-film architectures and detailed micrographs of $[C_2C_1im][OTf]$ films (50 ML) deposited on ITO/glass and carbon/ITO/glass surfaces. The morphology of the samples was assessed after 7 days, 4 days, and 2 days of film deposition. S10

Figure S10. Thin-film architectures and detailed micrographs of $[C_2C_1im][OTf]$ films (150 ML) deposited on ITO/glass and carbon/ITO/glass surfaces. The morphology of the samples was assessed after 7 days, 4 days, and 2 days of film deposition. S11

Figure S11. Thin-film architectures and detailed micrographs of thin films of $[C_2C_1im][NTf_2]$, $[C_8C_1im][NTf_2]$, $[C_2C_1im][OTf]$, and $[C_8C_1im][OTf]$ deposited on ITO/glass, carbon/ITO/glass, Ag/ITO/glass, and carbon/Ag//ITO/glass surfaces. S12

Figure S12. Thin-film architectures and detailed micrographs of thin films of $[C_2C_1im][NTf_2]$ and $[C_2C_1im][OTf]$ deposited on Au/ITO/glass and carbon/Au/ITO/glass surfaces. S13

Figure S13. Thin-film architectures and detailed micrographs of $[C_2C_1im][NTf_2]$ thin films deposited on Au/QC and carbon/Au/QC surfaces. S13

Figure S14. XPS survey spectra of the ITO/glass surface. S14

Figure S15. XPS survey spectra of the Au/ITO/glass surface. S14

Figure S16. XPS survey spectra of $[C_2C_1im][OTf]$ (100 ML) deposited on ITO/glass. S15

Figure S17. XPS survey spectra of $[C_2C_1im][OTf]$ (100 ML) deposited on Au/ITO/glass. S15

Figure S18. XPS survey spectra of [C_8C_1im][OTf] (100 ML) deposited on ITO/glass. S16

Figure S19. XPS survey spectra of [C_8C_1im][OTf] (100 ML) deposited on Au/ITO/glass. S16

Figure S20. High-resolution XPS spectra of [C_2C_1im][OTf] and [C_8C_1im][OTf] film surfaces deposited on ITO and Au. The XPS spectra were acquired for In 3d, Sn 3d, and Au 4f. S17

Figure S21. XPS survey spectra of the carbon (10 nm)/ITO/glass surface. S18

Figure S22. XPS survey spectra of the carbon (20 nm)/ITO/glass surface. S18

Figure S23. Thin-film architectures and detailed micrographs of [C_2C_1im][OTf] and [C_8C_1im][OTf] deposited on ITO/glass and carbon/ITO/glass. Each IL was deposited on ITO surfaces coated with varying amounts of carbon. S19

Table S1. Molar mass, density, viscosity, melting temperature, glass transition temperature, and superficial tension values for the ionic liquids [C_2C_1im][NTf_2], [C_8C1im][NTf_2], [C_2C_1im][OTf] and [C_8C1im][OTf]. S20

Table S2. Experimental conditions for the physical vapor deposition/thermal evaporation of each ionic liquid. Experimental variables related to the study of the influence of carbon on the morphology of different ionic liquids deposited at different amounts on the ITO/glass and C/ITO/glass surfaces. S20

Table S3. Experimental conditions for the physical vapor deposition/thermal evaporation of each ionic liquid. Experimental variables related to the study of the influence of carbon on the morphology of different ionic liquids deposited on the ITO/glass and C/ITO/glass, Ag/ITO/glass and C/Ag/ITO/glass surfaces, and Au/ITO/glass and C/Au/ITO/glass surfaces. S21

Table S4. Experimental conditions for the physical vapor deposition/thermal evaporation of [C_2C_1im][NTf_2]. Experimental variables related to the influence of carbon on the morphology of different ionic liquids deposited on the gold-coated quartz crystal (QC) and on the carbon-coated QC (C/QC). S21

Table S5. Experimental conditions for the physical vapor deposition/thermal evaporation of each ionic liquid. Experimental variables related to the study of the influence of carbon thickness on the morphology of different ionic liquids deposited on ITO/glass substrates. S22

Table S6. Experimental conditions for the physical vapor deposition/thermal evaporation of each ionic liquid. Experimental variables related to the time-dependent study on the morphology of different ionic liquids deposited on ITO/glass and C/ITO/glass substrates. S22

Table S7. Experimental C_{cation} : C_{anion}, N_{cation} : $F_{anion,\ and}$ N_{cation} : S_{anion} ratios derived from the XPS data. The expected/empiric values based on a cation-to-anion ratio of 1:1 are presented for comparison. S23

REFERENCES S24

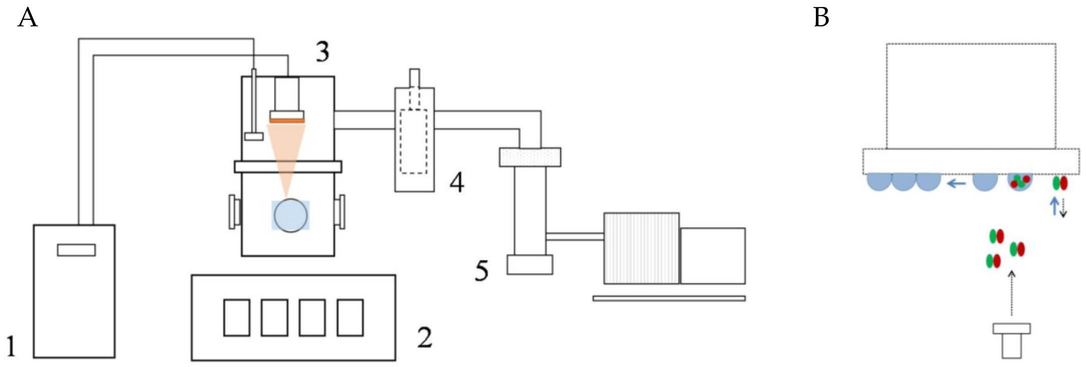

Figure S1. Schematic representation of the vacuum thermal evaporation methodology: (A) ThinFilmVD apparatus (1 – cooling system, 2 – instrumentation box, 3 – vacuum chamber, 4 – N_2 (l) metallic trap, 5 – vacuum pumping system); (B) schematic detail of the PVD of ionic liquids by thermal evaporation from a Knudsen cell.[1,2]

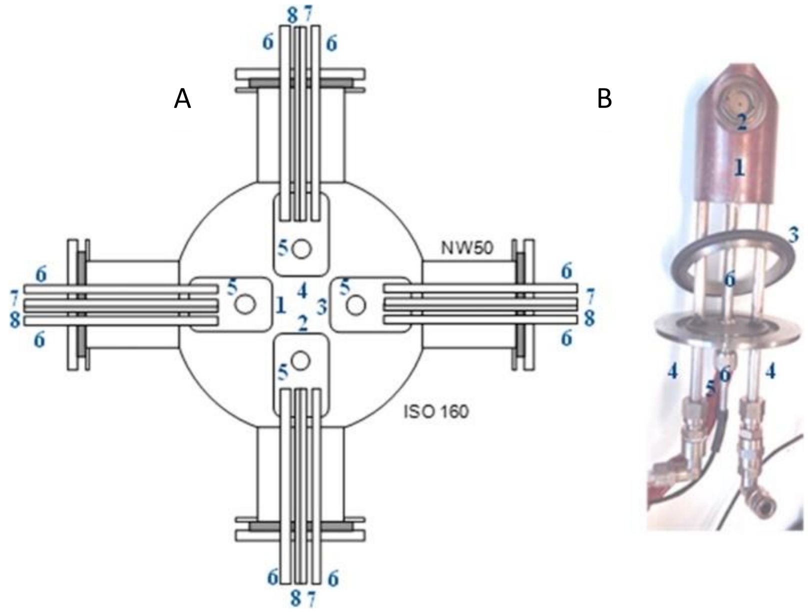

Figure S2. A – Schematic representation of the ovens of the ThinFilmVD apparatus: 1, 2, 3, 4 – individual copper ovens; 5 – cavity of the Knudsen cell screwing; 6 – air cooling tube; 7 – heater; 8, – Pt100 sensor; B – Image of an individual oven (top view): 1 – copper block; 2 – Knudsen cell; 3 – Viton O-ring; 4 – cooling system; 5 – heater; 6 – Pt100.[1]

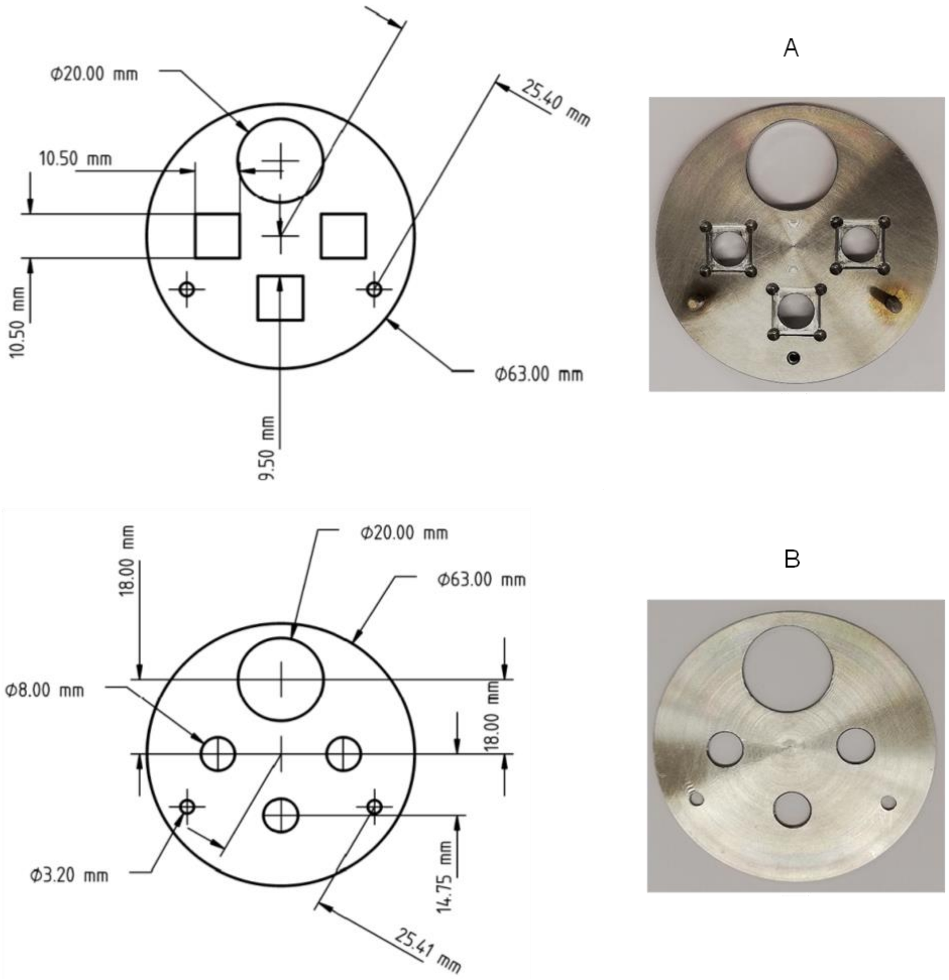

Figure S3. Schematic representation (left) and images (right) of the substrate support system. The support was used for the simultaneous deposition of each ionic liquid on "clean" and carbon-coated surfaces (ITO and C/ITO, Ag and C/ITO, Au and C/Au).

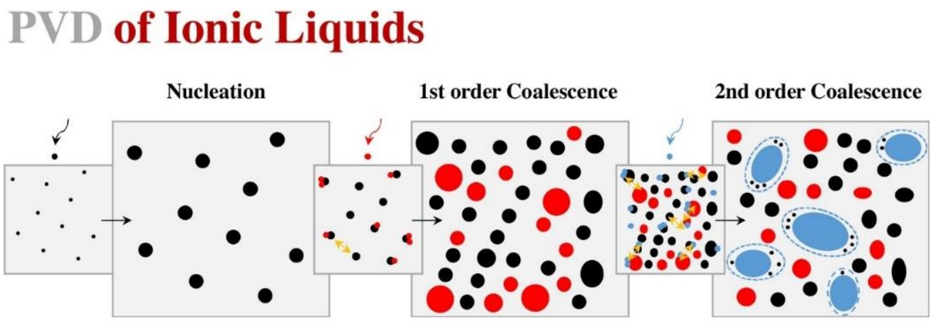

Figure S4. Schematic illustration of the typical mechanisms of nucleation and growth of ionic liquid films obtained by vapor deposition: minimum free area to promote nucleation (MFAN); first-order coalescence; second-order coalescence.[2]

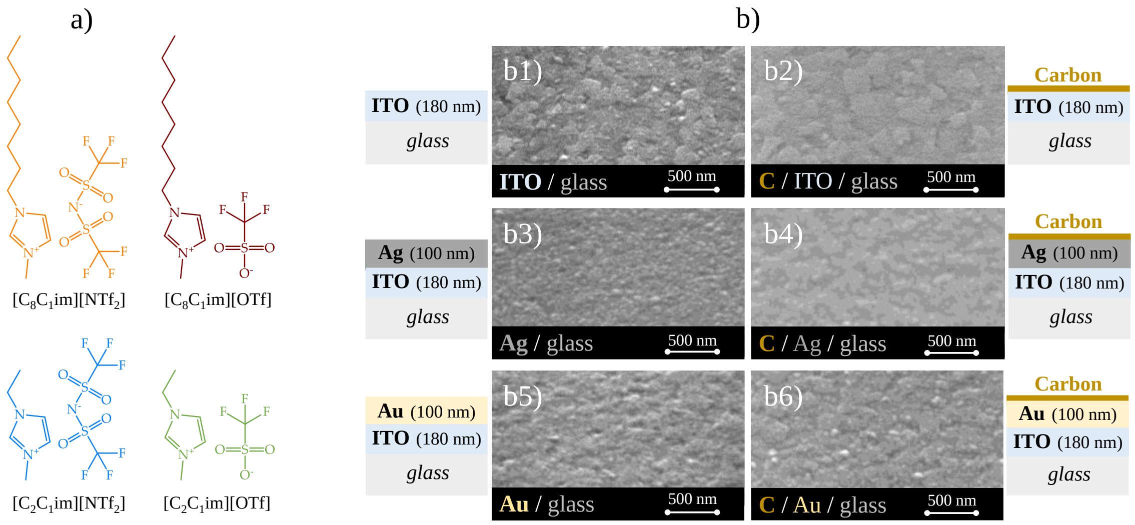

Figure S5. (a) Molecular structures of the ILs under study and their respective acronyms: 1-methyl-3-octylimidazolium bis(trifluoromethylsulfonyl)amide, [C_8C_1im][NTf_2]; 1-methyl-3-octylimidazolium triflate, [C_8C_1im][OTf]; 1-ethyl-3-methylimidazolium bis(trifluoromethylsulfonyl)amide, [C_2C_1im][NTf_2]; 1-ethyl-3-methylimidazolium triflate, [C_2C_1im][OTf]. (b) Thin-film architectures and detailed micrographs of the substrates: ITO/glass (b1); carbon/ITO/glass (b2); Ag/ITO/glass (b3); carbon/Ag/ITO/glass (b4); Au/ITO/glass (b5); carbon/Au/ITO/glass (b6). The carbon film was deposited with an approximate thickness of 20 nm. Micrographs were acquired at a lateral view of 45° with a magnification of 100,000×, using a high-resolution scanning electron microscope and employing a secondary electron detector.

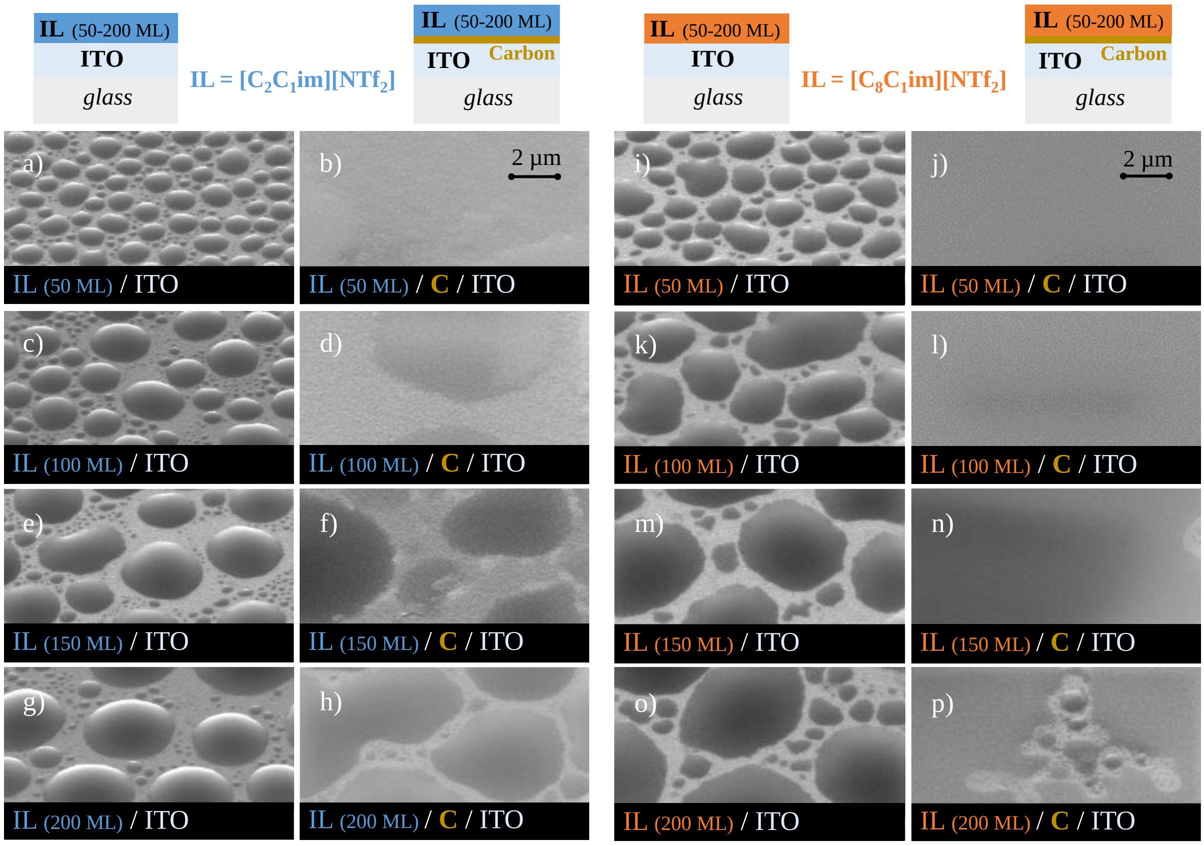

Figure S6. Thin-film architectures and detailed micrographs of thin films of $[C_2C_1im][NTf_2]$ (images a-h) and $[C_8C_1im][NTf_2]$ (images i-p) deposited on ITO/glass and carbon/ITO/glass surfaces (carbon thickness ≈ 20 nm). Each IL was deposited on both surfaces with varied thicknesses (50, 100, 150, and 200 ML). Micrographs were acquired at a lateral view of 45° with a magnification of 20,000×, using a high-resolution scanning electron microscope and employing a secondary electron detector.

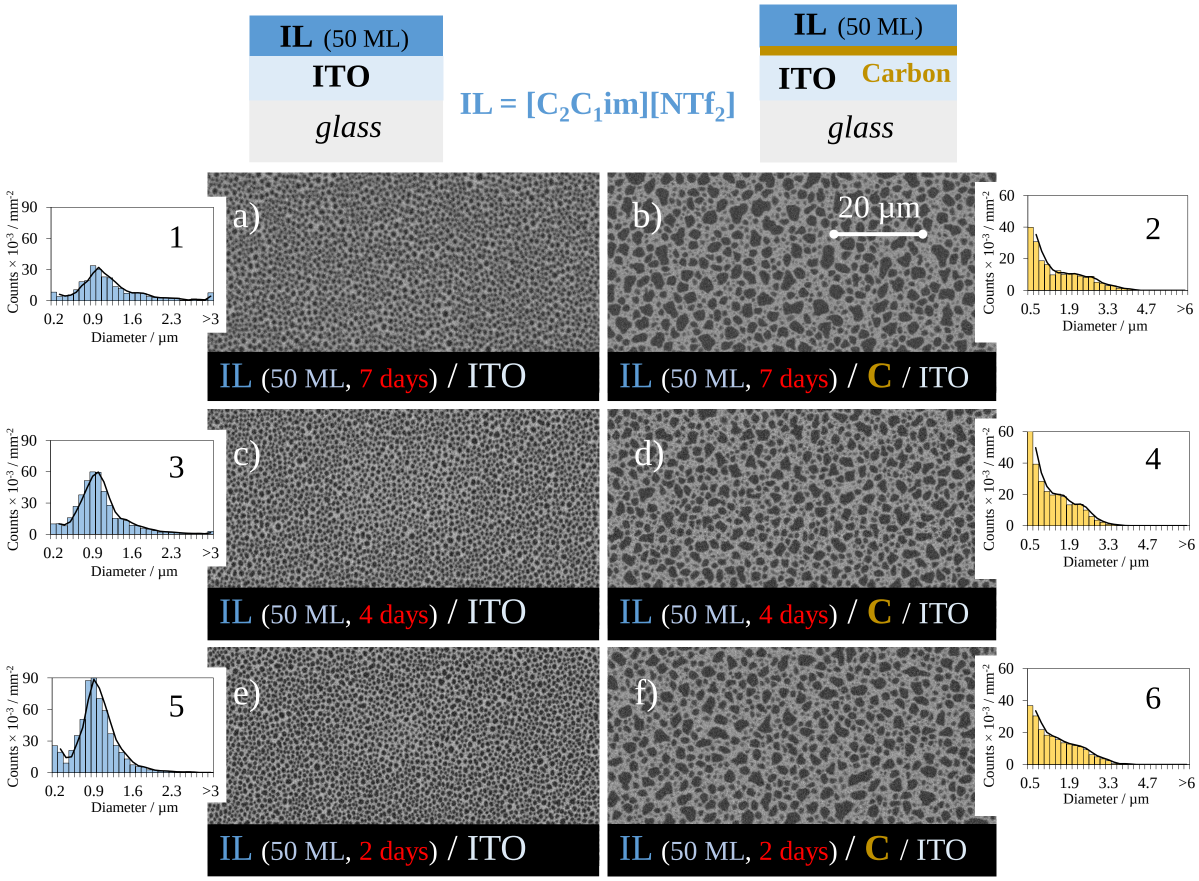

Figure S7. Thin-film architectures and detailed micrographs of $[C_2C_1im][NTf_2]$ films deposited on ITO/glass (images a, c, and e) and carbon/ITO/glass surfaces (images b, d, and f). The morphology of the samples was assessed after 7 days (images a and b), 4 days (images c and d), and 2 days (images e and f) of film deposition. Histograms 1-6 present the droplet size distribution obtained for each sample. Both films were deposited under similar experimental conditions with a thickness of 50 ML on both surfaces. Micrographs were acquired with a scanning electron microscope using a backscattered electron detector.

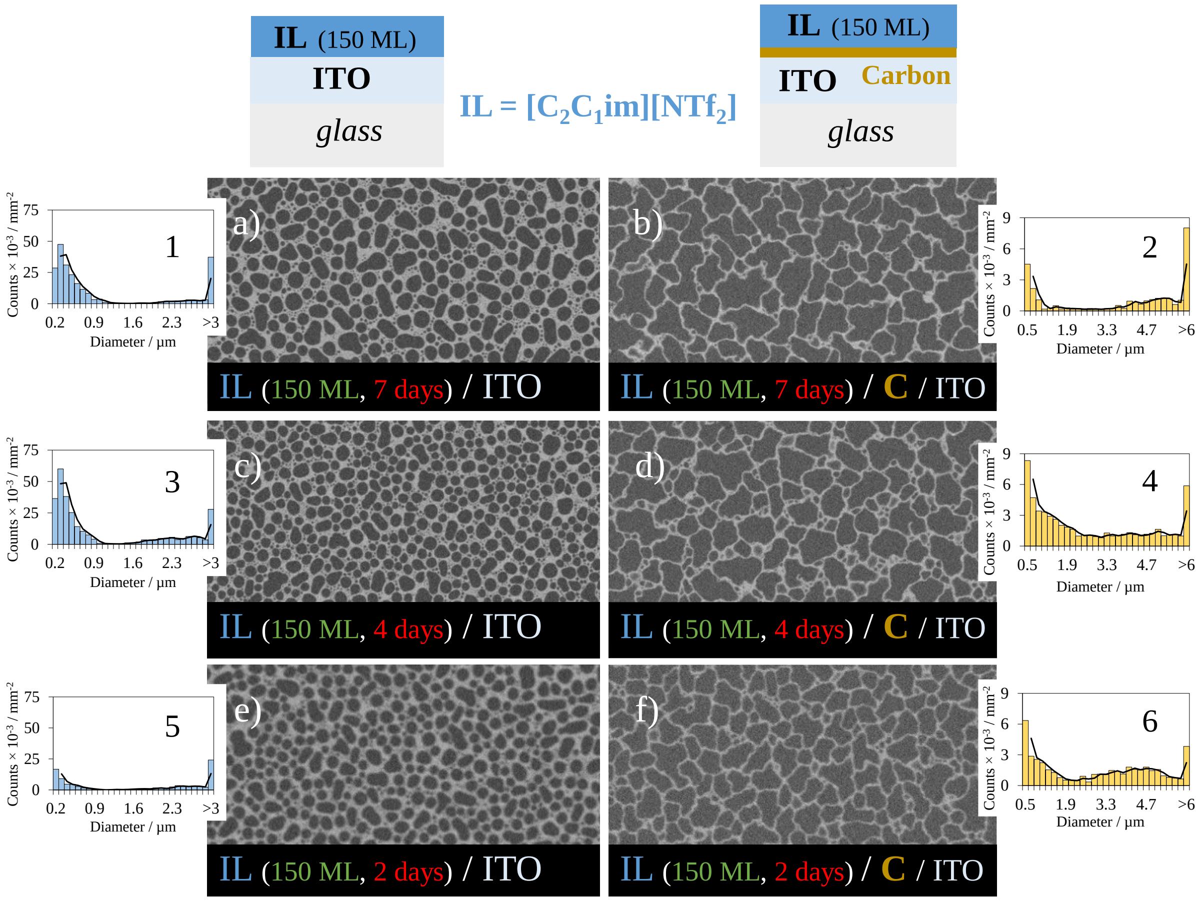

Figure S8. Thin-film architectures and detailed micrographs of $[C_2C_1im][NTf_2]$ films deposited on ITO/glass (images a, c, and e) and carbon/ITO/glass surfaces (images b, d, and f). The morphology of the samples was assessed after 7 days (images a and b), 4 days (images c and d), and 2 days (images e and f) of film deposition. Histograms 1-6 present the droplet size distribution obtained for each sample. Both films were deposited under similar experimental conditions with a thickness of 150 ML on both surfaces. Micrographs were acquired with a scanning electron microscope using a backscattered electron detector.

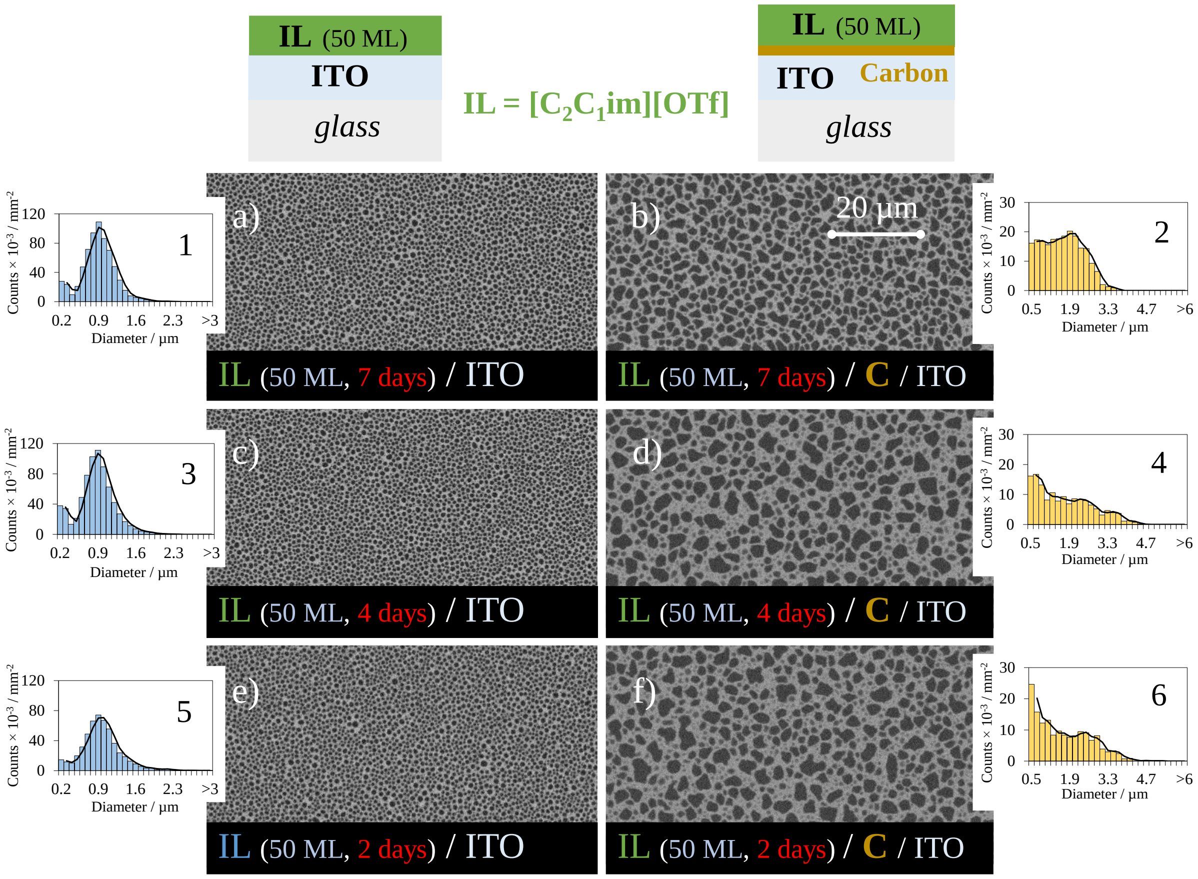

Figure S9. Thin-film architectures and detailed micrographs of $[C_2C_1im][OTf]$ films deposited on ITO/glass (images a, c, and e) and carbon/ITO/glass surfaces (images b, d, and f). The morphology of the samples was assessed after 7 days (images a and b), 4 days (images c and d), and 2 days (images e and f) of film deposition. Histograms 1-6 present the droplet size distribution obtained for each sample. Both films were deposited under similar experimental conditions with a thickness of 50 ML on both surfaces. Micrographs were acquired with a scanning electron microscope using a backscattered electron detector.

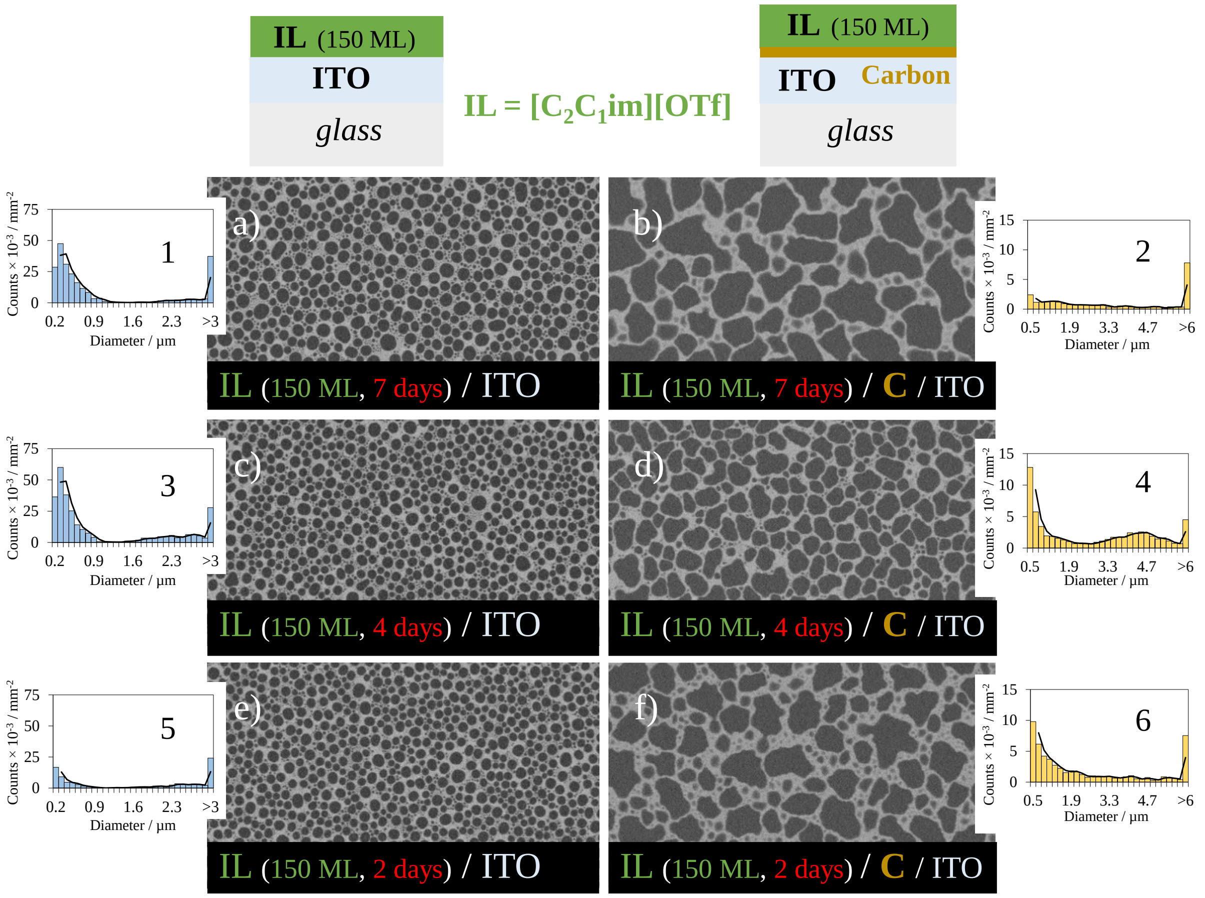

Figure S10. Thin-film architectures and detailed micrographs of $[C_2C_1im][OTf]$ films deposited on ITO/glass (images a, c, and e) and carbon/ITO/glass surfaces (images b, d, and f). The morphology of the samples was assessed after 7 days (images a and b), 4 days (images c and d), and 2 days (images e and f) of film deposition. Histograms 1-6 present the droplet size distribution obtained for each sample. Both films were deposited under similar experimental conditions with a thickness of 150 ML on both surfaces. Micrographs were acquired with a scanning electron microscope using a backscattered electron detector.

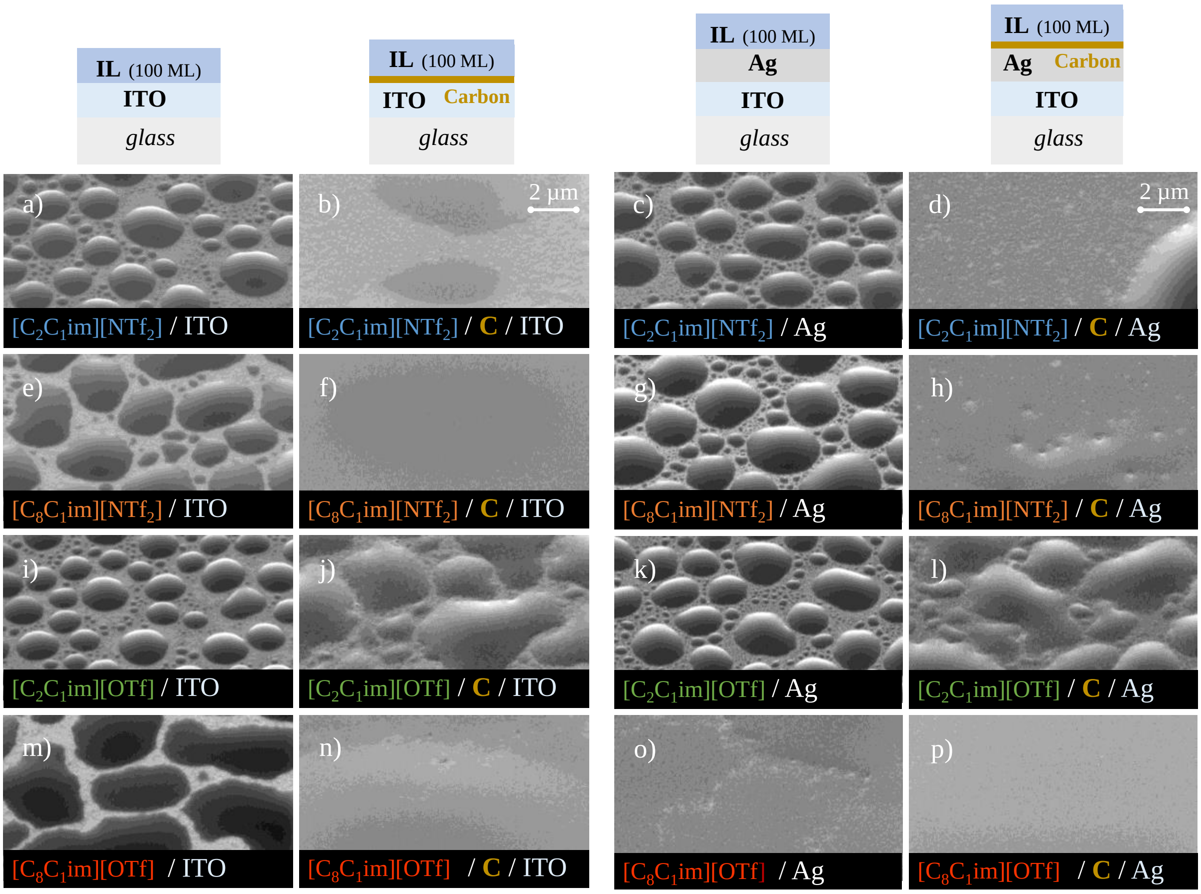

Figure S11. Thin-film architectures and detailed micrographs of thin films of $[C_2C_1im][NTf_2]$ (images a-d), $[C_8C_1im][NTf_2]$ (images e-h), $[C_2C_1im][OTf]$ (images i-l), and $[C_8C_1im][OTf]$ (images m-p) deposited on ITO/glass (first column), carbon/ITO/glass (second column), Ag/ITO/glass (third column), and carbon/Ag//ITO/glass (fourth column) surfaces (carbon thickness ≈ 20 nm). Each IL was deposited on both surfaces with a thickness of 100 ML. Micrographs were acquired at a lateral view of 45° with a magnification of 20,000×, using a high-resolution scanning electron microscope and employing a secondary electron detector.

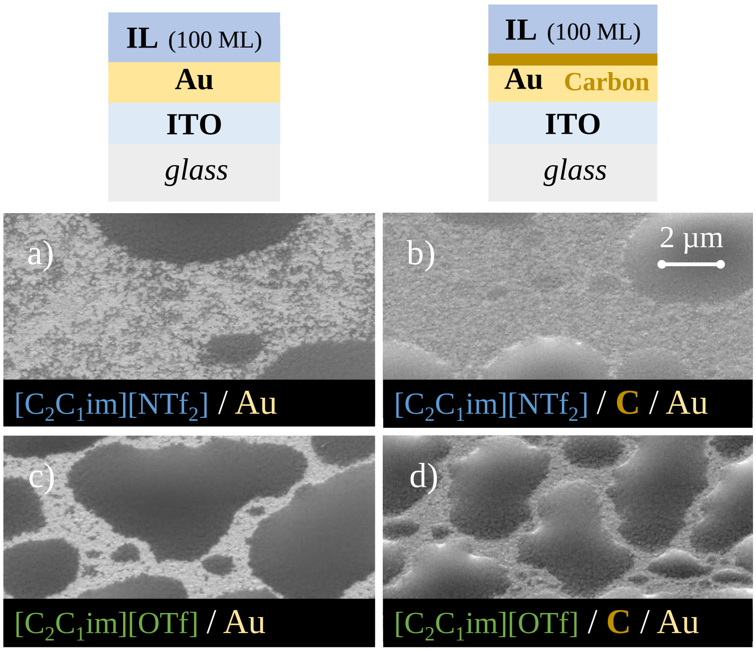

Figure S12. Thin-film architectures and detailed micrographs of thin films of $[C_2C_1im][NTf_2]$ (images a and b) and $[C_2C_1im][OTf]$ (images c and d) deposited on Au/ITO/glass (first column) and carbon/Au/ITO/glass (second column) surfaces (carbon thickness ≈ 20 nm). Each IL was deposited on both surfaces with a thickness of 100 ML. Micrographs were acquired at a lateral view of 45° with a magnification of 20,000×, using a high-resolution scanning electron microscope and employing a secondary electron detector.

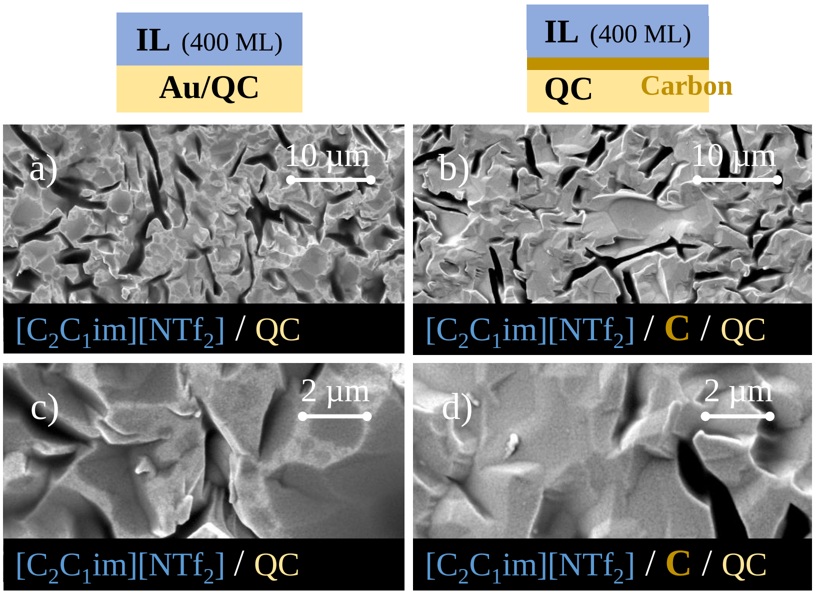

Figure S13. Thin-film architectures and detailed micrographs of $[C_2C_1im][NTf_2]$ thin films deposited on Au/QC (first column) and carbon/Au/QC (second column) surfaces (carbon thickness ≈ 20 nm). The IL was deposited on both surfaces with a thickness of 400 ML. Micrographs were acquired from a top view with magnifications of 5000× (images a and b) and 20,000× (images c and d), using a high-resolution scanning electron microscope and employing a secondary electron detector.

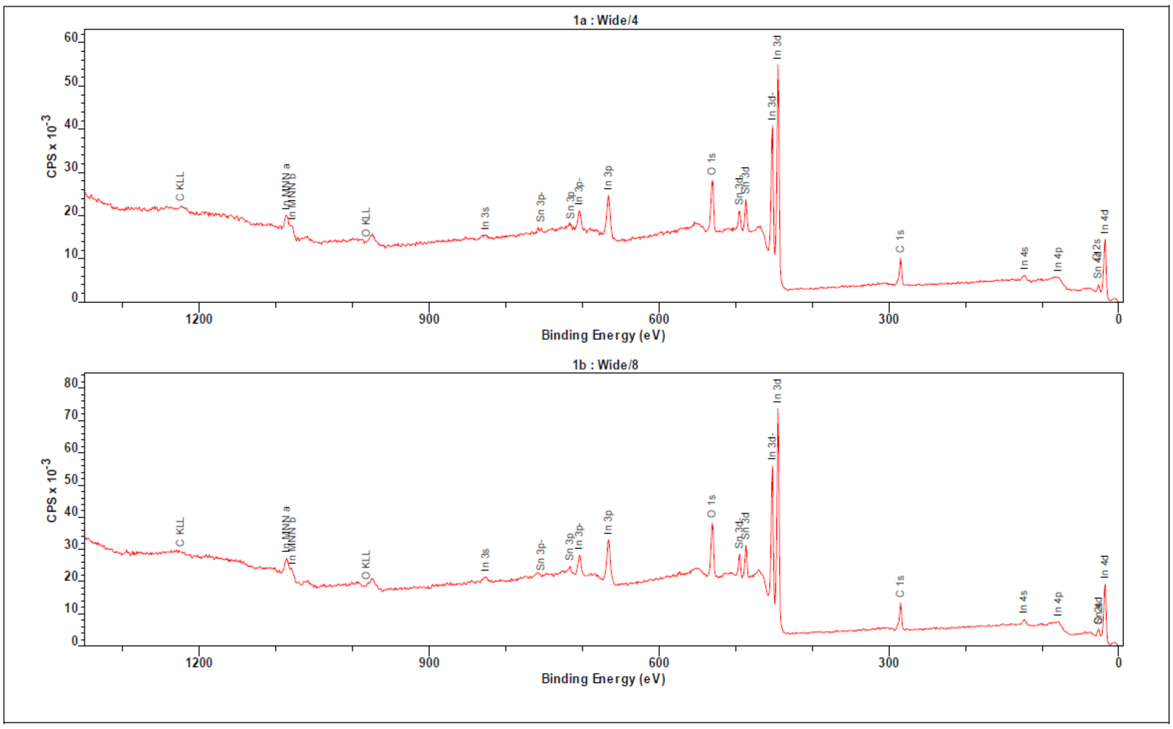

Figure S14. XPS survey spectra of the ITO/glass surface. The two spectra correspond to data obtained from the analysis of two different areas, each measuring 300 µm × 700 µm.

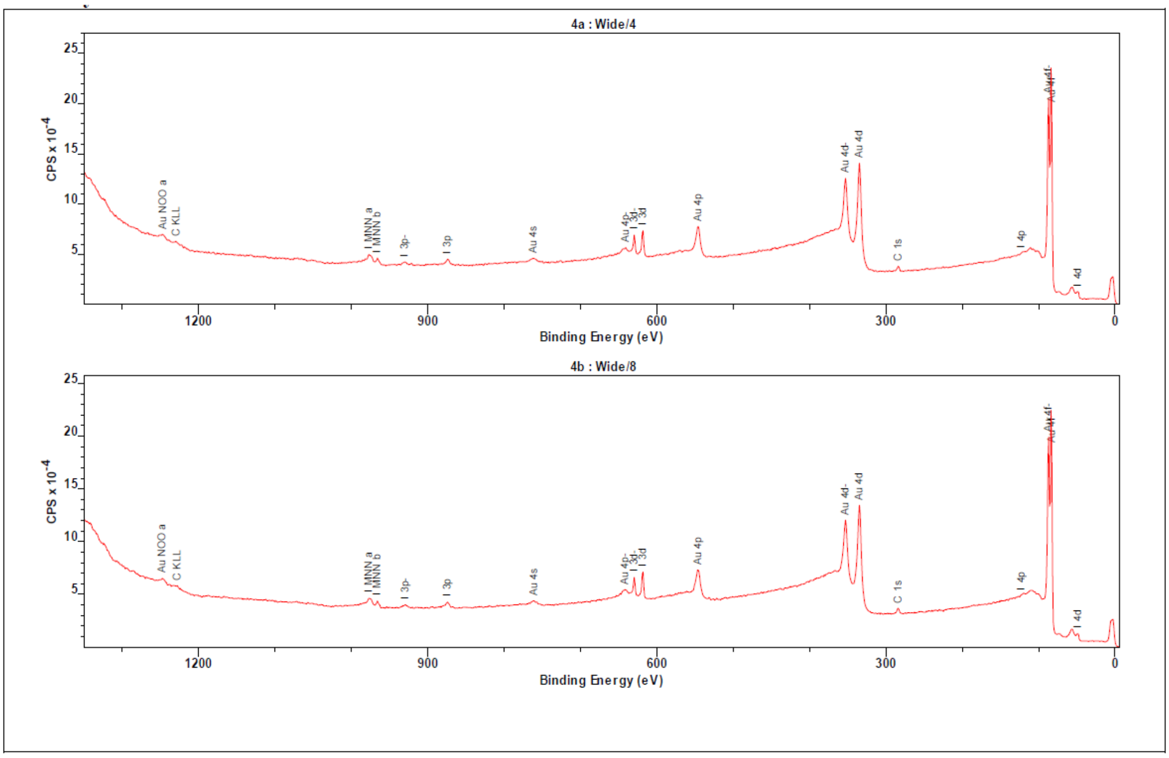

Figure S15. XPS survey spectra of the Au/ITO/glass surface. The two spectra correspond to data obtained from the analysis of two different areas, each measuring 300 µm × 700 µm.

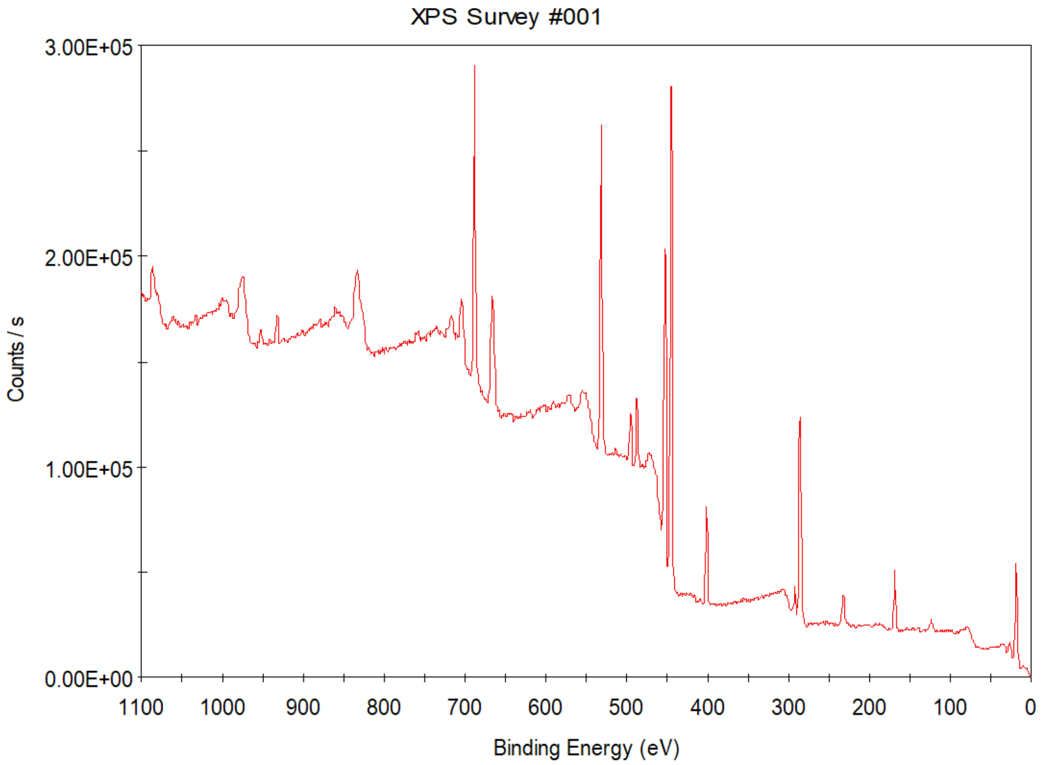

Figure S16. XPS survey spectra of [C_2C_1im][OTf] (100 ML) deposited on ITO/glass.

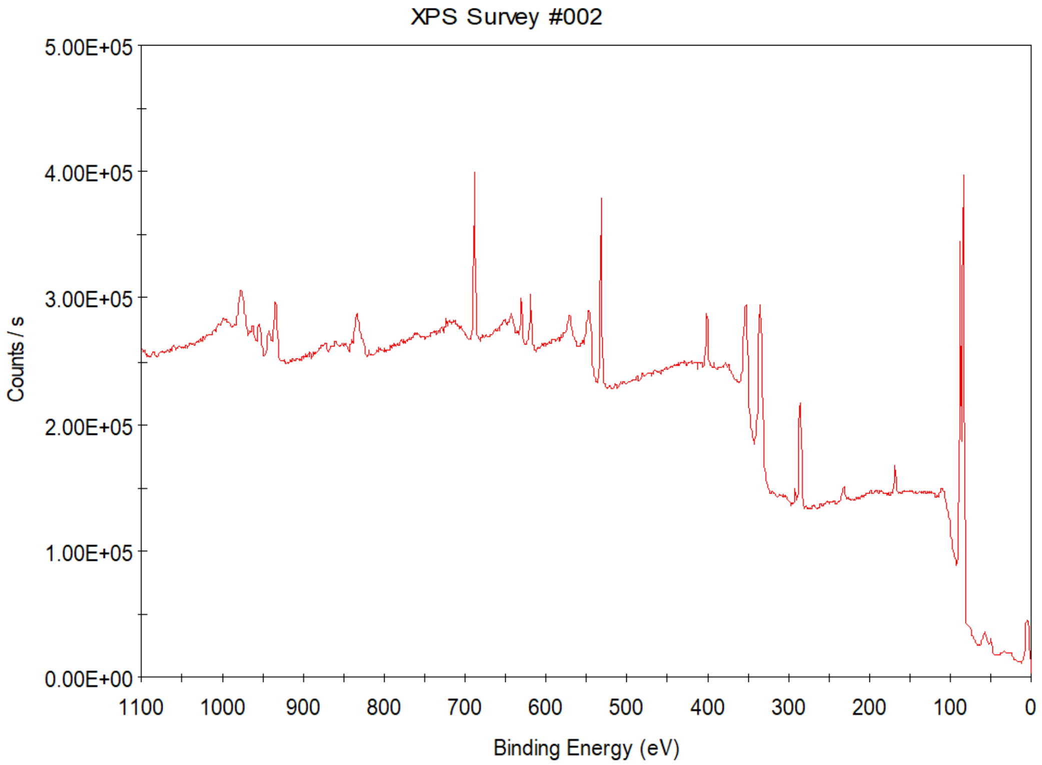

Figure S17. XPS survey spectra of [C_2C_1im][OTf] (100 ML) deposited on Au/ITO/glass.

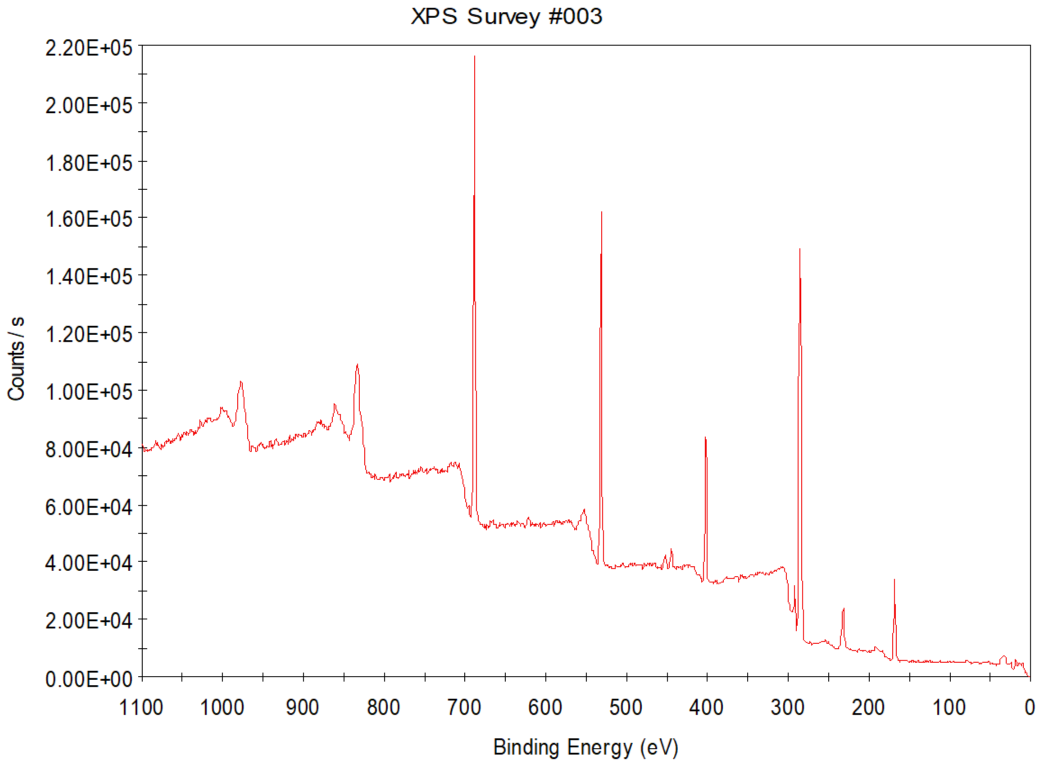

Figure S18. XPS survey spectra of [C_8C_1im][OTf] (100 ML) deposited on ITO/glass.

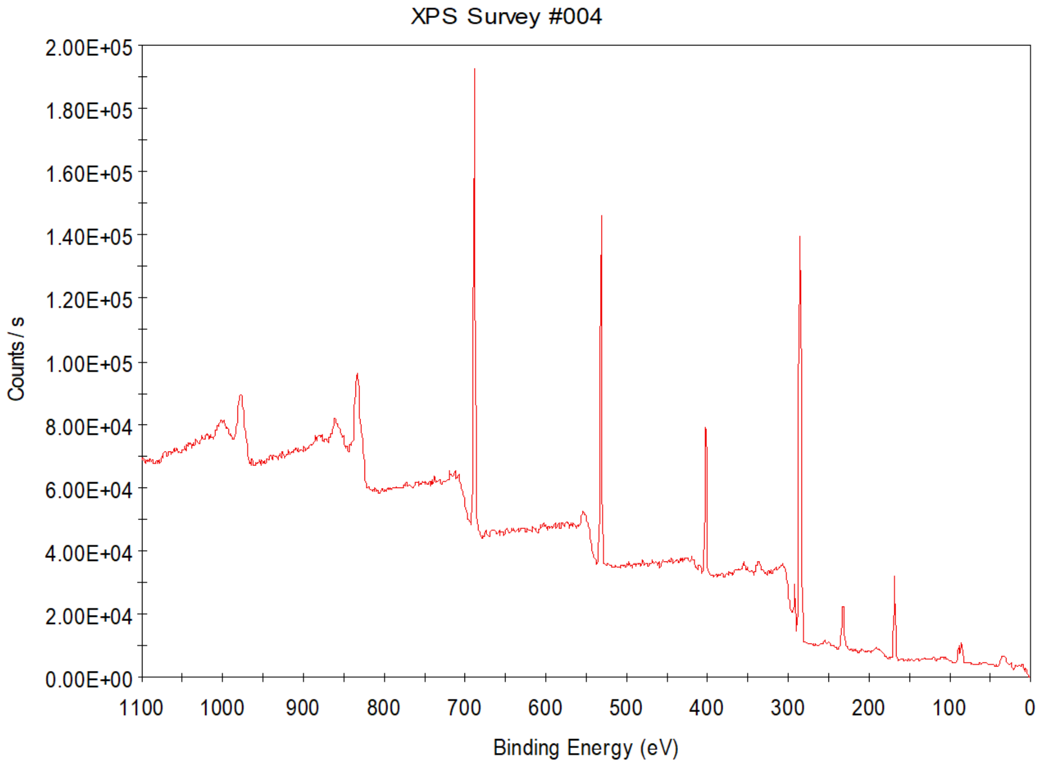

Figure S19. XPS survey spectra of [C_8C_1im][OTf] (100 ML) deposited on Au/ITO/glass.

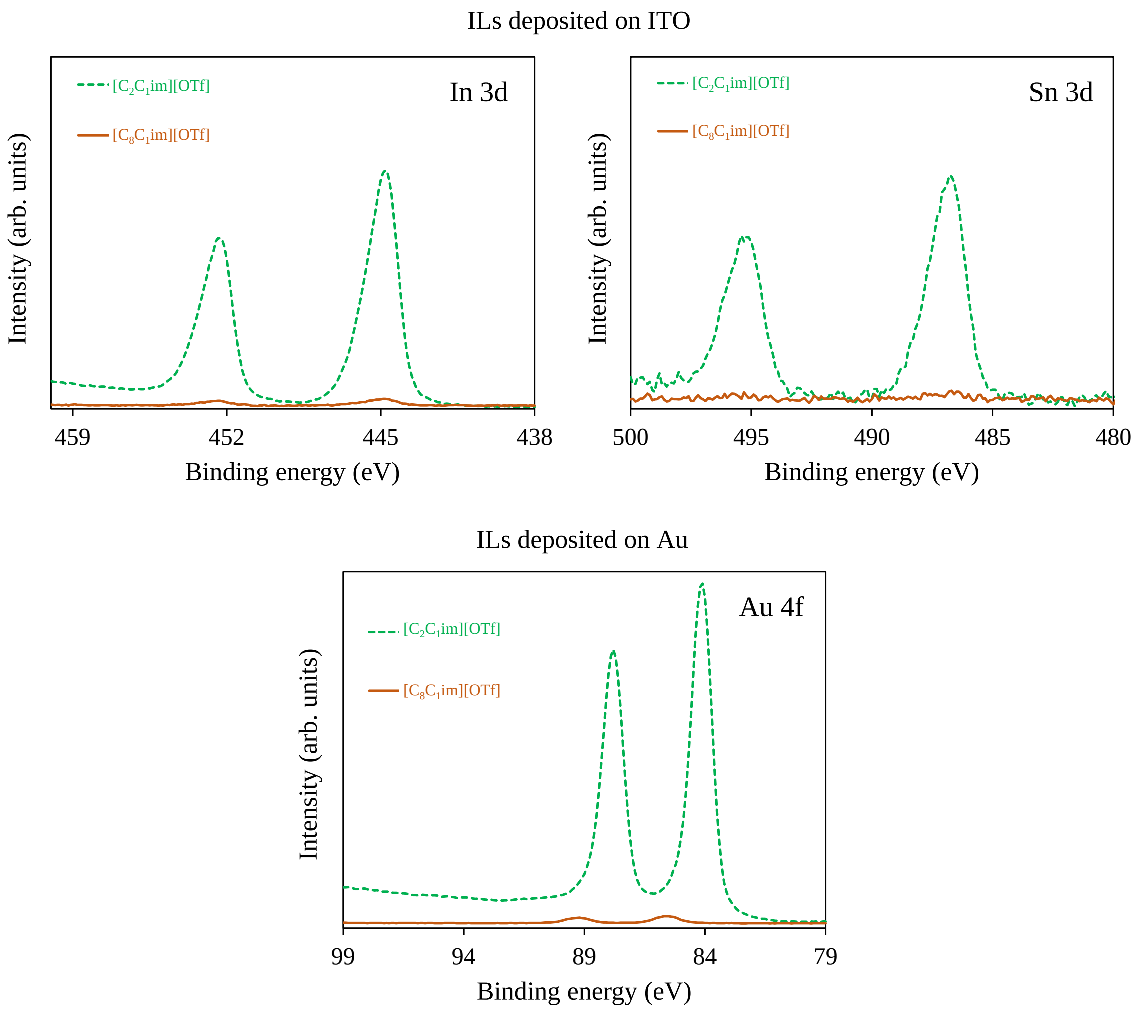

Figure S20. High-resolution XPS spectra of [C_2C_1im][OTf] (dashed lines) and [C_8C_1im][OTf] (solid lines) film surfaces deposited on ITO (spectra on the top) and Au (spectrum on the bottom). The XPS spectra were acquired for In 3d, Sn 3d, and Au 4f.

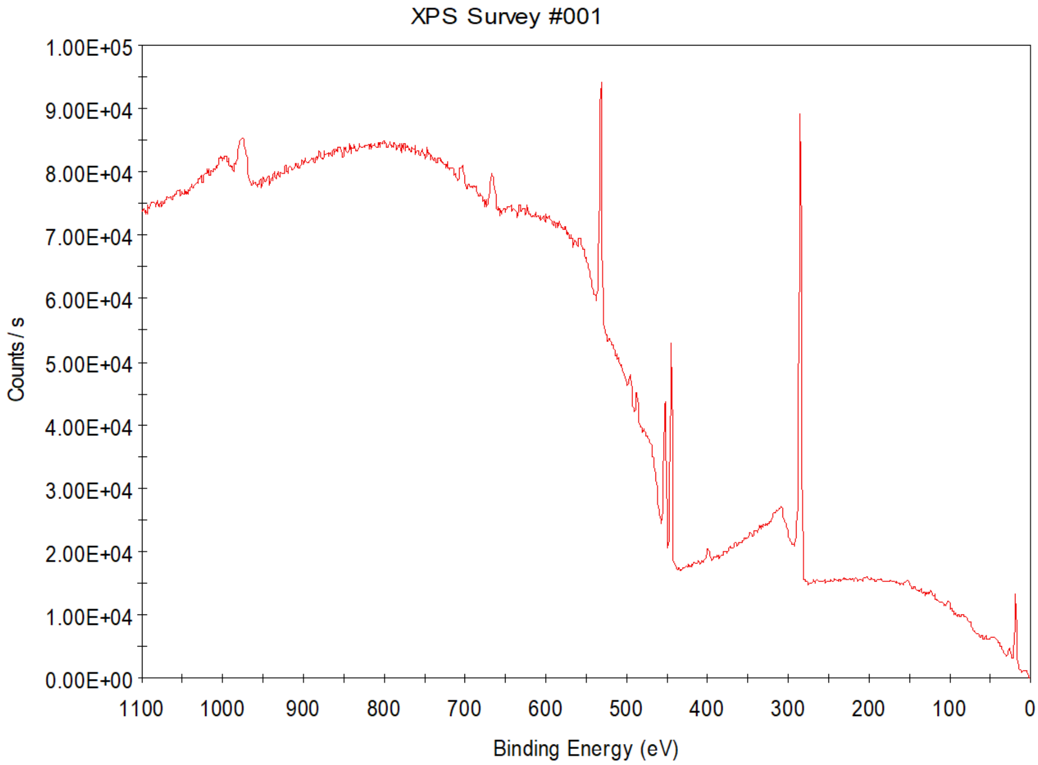

Figure S21. XPS survey spectra of the carbon (10 nm)/ITO/glass surface.

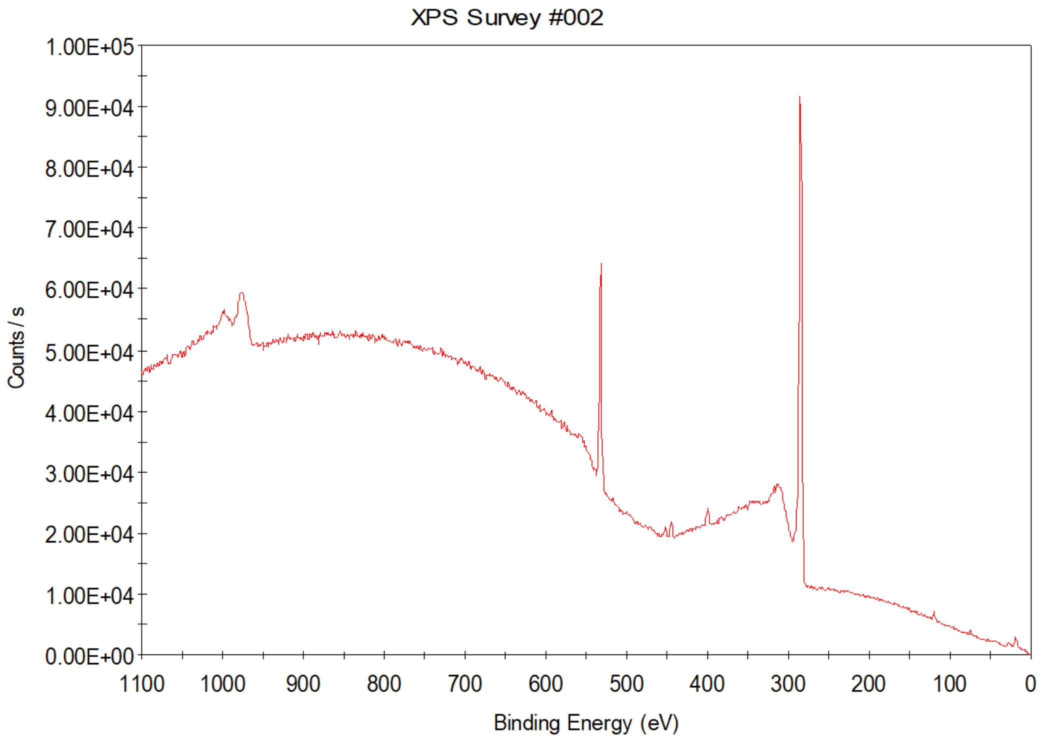

Figure S22. XPS survey spectra of the carbon (20 nm)/ITO/glass surface.

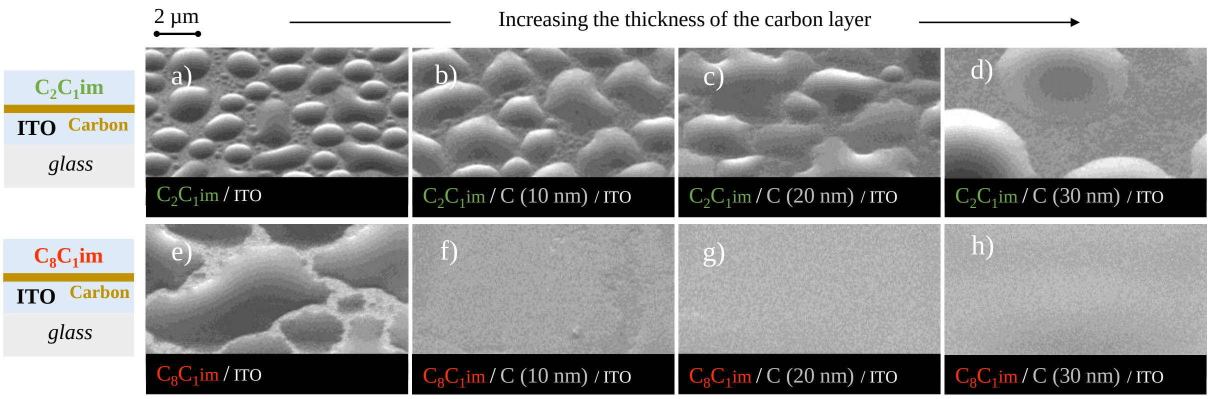

Figure S23. Thin-film architectures and detailed micrographs of [C_2C_1im][OTf] (images a-d) and [C_8C_1im][OTf] (images e-h) films (100 ML) deposited on ITO/glass (a and e) and carbon/ITO/glass (b, c, d, f, g, and h). Each IL was deposited on ITO surfaces coated with varying amounts of carbon: 0 nm (a and e); 10 nm (b and f); 20 nm (c and g); 30 nm (d and h). Micrographs were acquired at a lateral view of 45° with magnification of a 20,000× using a high-resolution scanning electron microscope and employing a secondary electron detector.

Table S1. Molar mass (M), density (ρ), viscosity (η), melting temperature (T_m), glass transition temperature (T_g), and superficial tension (γ) values for the ionic liquids $[C_2C_1im][NTf_2]$, $[C_8C1im][NTf_2]$, $[C_2C_1im][OTf]$ and $[C_8C1im][OTf]$.

Ionic Liquid	M / g·mol^{-1}	ρ (298K) / g·cm^{-3}	η / mPa·s	T_m / K	T_g / K	γ / mN·m^{-1}
$[C_2C_1im][NTf_2]$	391.31	1.52[3]<br>1.519[4,5]	33.0[9] (298K)	260[12]<br>255[13]<br>254[14]	178[12]<br>180[13]	36.9[5] (293K)<br>35.2[5] (298K)
$[C_8C_1im][NTf_2]$	475.47	1.31[6]	93.1[9] (298K)	250-264 [15]	185[15]	31.93[16] (293K)
$[C_2C_1im][OTf]$	260.23	1.38593[7] (297.94 K)	45.7[10] (298 K)	258[13]	175[13]	39.2[17] (298K)
$[C_8C_1im][OTf]$	344.40	1.1932[8]	127.4[11] (296.7 K)	-------	-------	28.5[17] (298K)

Table S2. Experimental conditions for the physical vapor deposition/thermal evaporation of each ionic liquid: effusion temperature ($T_{eff.}$); substrate temperature ($T_{subst.}$); equilibrium vapor pressure (EVP); orifice diameter of the Knudsen effusion cell; mass flow rate at the substrate surface [Φ (QCM)] and corresponding deposition rate in Å·s^{-1}; deposition time. Experimental variables related to the study of the influence of carbon on the morphology of different ionic liquids deposited at different amounts (different monolayers, ML) on the ITO/glass and C/ITO/glass surfaces (C = carbon).

Precursor	ML	$T_{eff.}$ K	$T_{subst.}$ K	EVP[a)] Pa	Orifice diameter mm	Φ (QCM) ng·cm^{-2}·s^{-1}	Deposition rate Å·s^{-1}	Deposition time s
			**$[C_nC_1im][NTf_2]$/ substrate** | substrates: ITO/glass; C/ITO/glass					
**$[C_2C_1im][NTf_2]$**	50	493.2	283.2	0.14	2.1	4.7	0.31 ± 0.02	1306
	100	491.2		0.13		4.9	0.32 ± 0.05	2512
	150	480.2		0.07		5.2	0.34 ± 0.07	3375
	200	498.2		0.19		5.6	0.37 ± 0.2	4260
**$[C_8C_1im][NTf_2]$**	50	501.2	283.2	0.14	2.1	4.1	0.31 ± 0.02	1500
	100	493.2		0.09		4.1	0.31 ± 0.02	2896
	150	498.2		0.12		3.9	0.30 ± 0.01	4331
	200	501.2		0.14		3.8	0.29 ± 0.05	5777

a) The EVP at each evaporation temperature was derived from literature data reporting volatility studies of the ILs: $[C_2C_1im][NTf_2]$ and $[C_8C_1im][NTf_2]$.[18]

Table S3. Experimental conditions for the physical vapor deposition/thermal evaporation of each ionic liquid: effusion temperature ($T_{eff.}$); substrate temperature ($T_{subst.}$); equilibrium vapor pressure (*EVP*); orifice diameter of the Knudsen effusion cell; mass flow rate at the substrate surface [Φ (QCM)] and corresponding deposition rate in Å·s^{-1}; deposition time. Experimental variables related to the study of the influence of carbon on the morphology of different ionic liquids deposited with 100 ML on the ITO/glass and C/ITO/glass, Ag/ITO/glass and C/Ag/ITO/glass surfaces, and Au/ITO/glass and C/Au/ITO/glass surfaces (C = carbon).

Precursor	Thickness	$T_{eff.}$	$T_{subst.}$	EVP	Orifice diameter	Φ (QCM)	Deposition rate	Deposition time
	ML	K	K	Pa	mm	ng·cm^{-2}·s^{-1}	Å·s^{-1}	s
**[$C_nC_1$im][anion] / substrate** | substrates: ITO/glass; C/ITO/glass								
**[C_2C_1im][NTf_2]**	100	491.2	283.2	0.13[a)]	2.1	4.9	0.32 ± 0.05	2512
**[C_8C_1im][NTf_2]**		493.2		0.09[a)]		4.1	0.31 ± 0.02	2896
**[C_2C_1im][OTf]**		543.2		[b)]		4.0	0.29 ± 0.02	2785
**[C_8C_1im][OTf]**		519.2		[b)]		3.5	0.29 ± 0.01	3080
**[$C_nC_1$im][anion]/ substrate** | substrates: Ag/ITO/glass; C/Ag/ITO/glass								
**[C_2C_1im][NTf_2]**	100	496.2	283.2	0.17[a)]	2.1	4.4	0.29 ± 0.02	2790
**[C_8C_1im][NTf_2]**		506.2		0.19[a)]		4.1	0.31 ± 0.01	3041
**[C_2C_1im][OTf]**		542.2		[b)]		3.9	0.28 ± 0.03	1306
**[C_8C_1im][OTf]**		505.2		[b)]		3.6	0.30 ± 0.02	2880
**[C_2C_1im][anion] / substrate** | substrates: Au/ITO/glass; C/Au/ITO/glass								
**[C_2C_1im][NTf_2]**	100	496.2	283.2	0.17[a)]	2.1	4.6	0.30 ± 0.01	2785
**[C_2C_1im][OTf]**		543.2		[b)]		4.0	0.29 ± 0.03	2867

[a)] The EVP at each evaporation temperature was derived from literature data reporting volatility studies of the ILs: [C_2C_1im][NTf_2] and [C_8C_1im][NTf_2].[18]

[b)] Accurate data for the EVP of [C_2C_1im][OTf] and [C_8C_1im][OTf] were not found elsewhere. Nevertheless, there are reports on the determination of vaporization enthalpies indicating the lower volatility of the [OTf]-based ILs in comparison to their congeners [NTf_2]-based ILs. In fact, in this work, at the same evaporation temperature lower deposition rates were observed for [OTf]-based. The EVP of [C_2C_1im][OTf] and [C_8C_1im][OTf] at the studied evaporation temperatures are estimated to be within the interval between 0.01 and 1 Pa. In addition, their EVP may be lower than observed for [C_2C_1im][NTf_2] and [C_8C_1im][NTf_2].

Table S4. Experimental conditions for the physical vapor deposition/thermal evaporation of [C_2C_1im][NTf_2]: effusion temperature ($T_{eff.}$); substrate temperature ($T_{subst.}$); equilibrium vapor pressure (*EVP*); orifice diameter of the Knudsen effusion cell; mass flow rate at the substrate surface [Φ (QCM)] and corresponding deposition rate in Å·s^{-1}; deposition time. Experimental variables related to the influence of carbon on the morphology of different ionic liquids deposited with 400 ML on the gold-coated quartz crystal (QC) and on the carbon-coated QC (C/QC) (C = carbon).

Substrate	Thickness	$T_{eff.}$	$T_{subst.}$	EVP[a)]	Orifice diameter	Φ (QCM)	Deposition rate	Deposition time
	ML	K	K	Pa	mm	ng·cm^{-2}·s^{-1}	Å·s^{-1}	s
**[C_2C_1im][NTf_2]/ substrate**								
QC	400	483.2	283.2	0.08	2.1	4.7	0.31 ± 0.05	9680
C/QC		480.2		0.07		4.6	0.30 ± 0.04	10165

[a)] The EVP at each evaporation temperature was derived from literature data reporting volatility studies of [C_2C_1im][NTf_2].[18]

Table S5. Experimental conditions for the physical vapor deposition/thermal evaporation of each ionic liquid: effusion temperature ($T_{eff.}$); substrate temperature ($T_{subst.}$); equilibrium vapor pressure (*EVP*); orifice diameter of the Knudsen effusion cell; mass flow rate at the substrate surface [Φ (QCM)] and corresponding deposition rate in Å·s^{-1}; deposition time. Experimental variables related to the study of the influence of carbon thickness on the morphology of different ionic liquids deposited with 100 ML on ITO/glass substrates (C = carbon).

Precursor	Substrate	$T_{eff.}$	$T_{subst.}$	EVP	Orifice diameter	Φ (QCM)	Deposition rate	Deposition time
		K	K	Pa	mm	ng·cm^{-2}·s^{-1}	Å·s^{-1}	s
				**[$C_nC_1$im][OTf] (100ML) / substrate**				
	ITO	537.2				4.0	0.29 ± 0.02	2660
	C(10 nm)	543.2				4.2	0.30 ± 0.02	2552
**[C_2C_1im][OTf]**	C(20 nm)	528.2	283	a)	2.1	4.3	0.31 ± 0.01	2340
	C(30 nm)	528.2				4.2	0.30 ± 0.03	2418
	Interface	531.2				4.3	0.31 ± 0.04	2300
	ITO	506.2				3.4	0.28 ± 0.02	2892
	C(10 nm)	525.2				3.5	0.29 ± 0.03	2785
**[C_8C_1im][OTf]**	C(20 nm)	503.2	283	a)	2.1	3.5	0.29 ± 0.01	2796
	C(30 nm)	510.2				3.4	0.28 ± 0.02	2876
	Interface	508.2				3.5	0.29 ± 0.02	2758

a) Accurate data for the EVP of [C_2C_1im][OTf] and [C_8C_1im][OTf] were not found elsewhere. Nevertheless, there are reports on the determination of vaporization enthalpies indicating the lower volatility of the [OTf]-based ILs in comparison to their congeners [NTf_2]-based ILs. In fact, in this work, at the same evaporation temperature lower deposition rates were observed for [OTf]-based. The EVP of [C_2C_1im][OTf] and [C_8C_1im][OTf] at the studied evaporation temperatures are estimated to be within the interval between 0.01 and 1 Pa. In addition, their EVP may be lower than observed for [C_2C_1im][NTf_2] and [C_8C_1im][NTf_2].

Table S6. Experimental conditions for the physical vapor deposition/thermal evaporation of each ionic liquid: storage time before morphological characterization; effusion temperature ($T_{eff.}$); substrate temperature ($T_{subst.}$); equilibrium vapor pressure (*EVP*); orifice diameter of the Knudsen effusion cell; mass flow rate at the substrate surface [Φ (QCM)] and corresponding deposition rate in Å·s^{-1}; deposition time. Experimental variables related to the time-dependent study on the morphology of different ionic liquids deposited with 50 and 150 ML on ITO/glass and C/ITO/glass substrates (C = carbon).

Precursor	Days[a)]	$T_{eff.}$	$T_{subst.}$	EVP	Orifice diameter	Φ (QCM)	Deposition rate	Deposition time
		K	K	Pa	mm	ng·cm^{-2}·s^{-1}	Å·s^{-1}	s
				**[C_2C_1im][NTf_2] / substrate**				
**[C_2C_1im][NTf_2] 50 ML**	7	488.2		0.11[b)]		5.0	0.33 ± 0.04	1237
	4	496.2		0.17[b)]		5.0	0.33 ± 0.02	1315
	2	513.2	283.2	0.42[b)]	2.1	5.5	0.36 ± 0.12	1540
**[C_2C_1im][NTf_2] 150 ML**	7	494.2		0.15[b)]		5.2	0.34 ± 0.07	3405
	4	493.2		0.14[b)]		4.7	0.31 ± 0.01	3782
	2	513.2		0.42[b)]		4.6	0.30 ± 0.02	4076
				**[C_2C_1im][OTf] / substrate**				
**[C_2C_1im][OTf] 50 ML**	7	498.2				4.2	0.30 ± 0.10	1290
	4	528.2				4.3	0.31 ± 0.03	1394
	2	521.2	283.2	c)	2.1	3.8	0.27 ± 0.03	1527
**[C_2C_1im][OTf] 150 ML**	7	496.2				4.4	0.32 ± 0.03	3240
	4	523.2				4.3	0.31 ± 0.08	3720
	2	521.2				4.2	0.30 ± 0.02	3685

a) storage time (days) before morphological characterization.
b) The EVP at each evaporation temperature was derived from literature data reporting volatility studies of [C_2C_1im][NTf_2].[18]
c) Accurate data for the EVP of [C_2C_1im][OTf] were not found elsewhere. Nevertheless, there are reports on the determination of vaporization enthalpies indicating the lower volatility of the [OTf]-based ILs in comparison to their congeners [NTf_2]-based ILs. In fact, in this work, at the same evaporation temperature lower deposition rates were observed for [OTf]-based. The EVP of [C_2C_1im][OTf] at the studied evaporation temperatures is estimated to be within the interval between 0.01 and 1 Pa. In addition, their EVP may be lower than observed for [C_2C_1im][NTf_2].

Table S7. Experimental C_{cation} : C_{anion}, N_{cation} : $F_{anion,}$ and N_{cation} : S_{anion} ratios derived from the XPS data. Values based on a cation-to-anion ratio of 1:1 are presented for comparison.

Substrate	Ionic liquid	Empirical Formula	C_{cation} : C_{anion}	N_{cation} : F_{anion}	N_{cation} : S_{anion}
ITO	[C_2C_1im][OTf]	$C_7H_{11}F_3N_2O_3S$	10 : 1[a)]<br>6 : 1 (empiric)	1.6 : 3<br>2 : 3 (empiric)	1.9 : 1<br>2 : 1 (empiric)
	[C_8C_1im][OTf]	$C_{13}H_{23}F_3N_2O_3S$	12.2 : 1<br>12 : 1 (empiric)	1.7 : 3<br>2 : 3 (empiric)	1.8 : 1<br>2 : 1 (empiric)
Au	[C_2C_1im][OTf]	$C_7H_{11}F_3N_2O_3S$	8.2 : 1[a)]<br>6 : 1 (empiric)	1.6 : 3<br>2 : 3 (empiric)	2.1 : 1<br>2 : 1 (empiric)
	[C_8C_1im][OTf]	$C_{13}H_{23}F_3N_2O_3S$	12.4 : 1<br>12 : 1 (empiric)	1.7 : 3<br>2 : 3 (empiric)	1.9 : 1<br>2 : 1 (empiric)

[a)] These values differ from the empirical ratio as they also take into account the substrate information, especially the presence of adventitious carbon contamination.

REFERENCES

(1) Costa, J. C. S.; Rocha, R. M.; Vaz, I. C. M.; Torres, M. C.; Mendes, A.; Santos, L. M. N. B. F. Description and Test of a New Multilayer Thin Film Vapor Deposition Apparatus for Organic Semiconductor Materials. *J. Chem. Eng. Data* **2015**, *60*, 3776–3791.

(2) Costa, J. C. S.; Coelho, A. F. S. M. G.; Mendes, A.; Santos, L. M. N. B. F. Nucleation and Growth of Microdroplets of Ionic Liquids Deposited by Physical Vapor Method onto Different Surfaces. *Appl. Surf. Sci.* **2018**, *428*, 242–249.

(3) Almeida, H. F. D.; Freire, M. G.; Fernandes, A. M.; Lopes-da-Silva, J. A.; Morgado, P.; Shimizu, K.; Filipe, E. J. M.; Canongia Lopes, J. N.; Santos, L. M. N. B. F.; Coutinho, J. A. P. Cation Alkyl Side Chain Length and Symmetry Effects on the Surface Tension of Ionic Liquids. *Langmuir* **2014**, *30*, 6408–6418.

(4) Rocha, M. A. A.; Neves, C. M. S. S.; Freire, M. G.; Russina, O.; Triolo, A.; Coutinho, J. A. P.; Santos, L. M. N. B. F. Alkylimidazolium Based Ionic Liquids: Impact of Cation Symmetry on Their Nanoscale Structural Organization. *J. Phys. Chem. B* **2013**, *117*, 10889–10897.

(5) Campos, R. M.; Alves, A. C. P. M.; Lima, M. A. L.; Farinha, A. F. M.; Cardoso, J. P. S.; Mendes, A.; Costa, J. C. S.; Santos, L. M. N. B. F. Morphology, Structure, and Dynamics of Pentacene Thin Films and Their Nanocomposites with [C_2C_1Im][NTf_2] and [C_2C_1Im][OTF] Ionic Liquids. *ChemPhysChem* **2020**, *21*, 1814–1825.

(6) Lexow, M.; Maier, F.; Steinrück, H.-P. Ultrathin Ionic Liquid Films on Metal Surfaces: Adsorption, Growth, Stability and Exchange Phenomena. *Adv. Phys. X* **2020**, *5*, 1761266.

(7) Klomfar, J.; Součková, M.; Pátek, J. Temperature Dependence Measurements of the Density at 0.1 MPa for 1-Alkyl-3-Methylimidazolium-Based Ionic Liquids with the Trifluoromethanesulfonate and Tetrafluoroborate Anion. *J. Chem. Eng. Data* **2010**, *55*, 4054–4057.

(8) Nebig, S.; Gmehling, J. Measurements of Different Thermodynamic Properties of Systems Containing Ionic Liquids and Correlation of These Properties Using Modified UNIFAC (Dortmund). *Fluid Ph. Equilibria* **2010**, *294*, 206–212.

(9) Tariq, M.; Carvalho, P.; Coutinho, P.; Marrucho, I. M.; Canongia Lopes, J. N.; Paulo, L. P. Viscosity of (C2–C14) 1-Alkyl-3-Methylimidazolium Bis(Trifluoromethylsulfonyl)Amide Ionic Liquids in an Extended Temperature Range. *Fluid Ph. Equilibria* **2011**, *301*, 22–32.

(10) Anwar, N.; Riyazuddeen. Excess Molar Volumes, Excess Molar Isentropic Compressibilities, Viscosity Deviations, and Activation Parameters for 1-Ethyl-3-Methyl-Imidazolium Trifluoro-Methanesulfonate + Dimethyl Sulfoxide And/or Acetonitrile at T = 298.15 to 323.15 K and P = 0.1 MPa. *J. Chem. Eng. Data* **2018**, *63*, 269–289.

(11) Diejomaoh Abafe, O. T.; Azim, M. M.; Martincigh, B. S.; Stark, A. Cation-Fluorinated Ionic Liquids: Synthesis, Physicochemical Properties and Comparison with Non-Fluorinated Analogues. *J. Mol. Liq.* **2022**, *349*, 118104.

(12) Rodrigues, A. S. M. C.; Santos, L. M. N. B. F., Nanostructuration Effect on the Thermal Behavior of Ionic Liquids. *ChemPhysChem* **2016**, *17*, 1512–1517.

(13) Every, H.; Bishop, A. G.; Forsyth, M.; MacFarlane, D. R. Ion Diffusion in Molten Salt Mixtures. *Electrochim. Acta* **2000**, *45*, 1279–1284.

(14) Krossing, I.; Slattery, J. M.; Daguenet, C.; Dyson, P. J.; Oleinikova, A.; Weingärtner, H. Why Are Ionic Liquids Liquid? A Simple Explanation Based on Lattice and Solvation Energies. *J. Am. Chem. Soc.* **2006**, *128*, 13427–13434.

(15) Paulechka, Y. U.; Blokhin, A. V.; Kabo, G. J.; A.A. Strechan. Thermodynamic Properties and Polymorphism of 1-Alkyl-3-Methylimidazolium Bis(Triflamides). *J. Chem. Thermodyn.* **2007**, *39*, 866–877.

(16) Carvalho, P.; Freire, M. G.; Marrucho, I. M.; Queimada, A. J.; Coutinho, P. Surface Tensions for the 1-Alkyl-3-Methylimidazolium Bis(Trifluoromethylsulfonyl)Imide Ionic Liquids. *J. Chem. Eng. Data* **2008**, *53*, 1346–1350.

(17) Tariq, M.; Freire, M. G.; Saramago, B.; Coutinho, J. A. P.; Lopes, J. N. C.; Rebelo, L. P. N. Surface Tension of Ionic Liquids and Ionic Liquid Solutions. *Chem. Soc. Rev.* **2012**, *41*, 829–868.

(18) Rocha, M. A. A., Lima, C. F. R. A. C., Gomes, L. R., Schröder, B., Coutinho, J. A. P., Marrucho, I. M.; Esperança, J. M. S. S.; Rebelo, L. P. N.; Shimizu, K.; Canongia Lopes, J. N.; Santos, L. M. N. B. F. High-Accuracy Vapor Pressure Data of the Extended [$C_nC_1$im][Ntf_2] Ionic Liquid Series: Trend Changes and Structural Shifts. *J. Phys. Chem. B* **2011**, *115*, 10919–10926.
